# Lanthanide-Doped Luminescent Nanophosphors *via* Ionic Liquids

**DOI:** 10.3389/fchem.2021.715531

**Published:** 2021-08-27

**Authors:** Rahul Kumar Sharma, Pushpal Ghosh

**Affiliations:** ^1^ Department of Chemistry, Government Shyam Sundar Agrawal PG College, Jabalpur, India; ^2^ Department of Chemistry, School of Chemical Sciences and Technology, Dr. Hari Singh Gour University (A Central University), Sagar, India

**Keywords:** nanophosphors, ionic liquid, rare-earth, photonic, bio-photonic

## Abstract

Lanthanide (Ln^3+^) ion(s)-doped or rare-earth ion(s)-doped nanomaterials have been considered a very important class of nanophosphors for various photonic and biophotonic applications. Unlike semiconductors and organic-based luminescent particles, the optical properties of Ln^3+^-doped nanophosphors are independent of the size of the nanoparticles. However, by varying the crystal phase, morphology, and lattice strain of the host materials along with making core-shell structure, the relaxation dynamics of dopant Ln^3+^ ions can be effectively tuned. Interestingly, a judicious choice of dopant ions leads to unparallel photophysical dynamics, such as quantum cutting, upconversion, and energy transfer. Recently, ionic liquids (ILs) have drawn tremendous attention in the field of nanomaterials synthesis due to their unique properties like negligible vapor pressure, nonflammability, and, most importantly, tunability; thus, they are often called “green” and “designer” solvents. This review article provides a critical overview of the latest developments in the ILs-assisted synthesis of rare-earth-doped nanomaterials and their subsequent photonic/biophotonic applications, such as energy-efficient lighting and solar cell applications, photodynamic therapy, and *in vivo* and *in vitro* bioimaging. This article will emphasize how luminescence dynamics of dopant rare-earth ions can be tuned by changing the basic properties of the host materials like crystal phase, morphology, and lattice strain, which can be eventually tuned by various properties of ILs such as cation/anion combination, alkyl chain length, and viscosity. Last but not least, different aspects of ILs like their ability to act as templating agents, solvents, and reaction partners and sometimes their “three-in-one” use in nanomaterials synthesis are highlighted along with various photoluminescence mechanisms of Ln^3+^ ion like up- and downconversion (UC and DC).

## Introduction

Lanthanide (Ln^3+^)-doped nanophosphors materials have gained appreciable attention for the development of nanotechnology due to their unprecedented applications in various fields, such as optoelectronic, magnetic, imaging, and solar cell applications (Jaque and Vetrone, 2012; Gai et al., 2014; Goldschmidt and Fischer, 2015; Sharma et al., 2017a; Qin et al., 2017; Runowski, 2020). These applications are fundamentally dependent on the doping of the Ln^3+^ ions because judicious doping of the Ln^3+^ ions in a suitable host material results in numerous photophysical processes such as energy transfer, upconversion, and downconversion (Gai et al., 2014; Goldschmidt and Fischer, 2015; Sharma et al., 2017a; Qin et al., 2017). Unlike photophysical processes observed for the semiconductors and organic nanomaterials, Ln^3+^-doped nanophosphors exhibit size-independent photophysical processes. However, their luminescence intensity can be tuned by varying the crystal phase of host materials, lattice strain, and morphology and making core-shell structures (Gai et al., 2014; Sharma et al., 2017a). Therefore, several host materials have been explored for doping of Ln^3+^ ions, including alkali/alkaline/lanthanide-based binary/ternary fluorides, lanthanide orthophosphates (LnPO_4_), and oxides (Ln_2_O_3_) (Guo et al., 2010; He et al., 2012; Jiang et al., 2012; Alammar et al., 2016; Cybińska et al., 2016; Sharma et al., 2017a; Zhao et al., 2018). In order to prepare the aforementioned host materials, numbers of synthesis methods, such as sol-gel, thermal decomposition, hydrothermal/solvothermal, microwave-assisted, sonochemical-assisted techniques, should be readily employed (He et al., 2011a; Ju and Mudring, 2013; Gai et al., 2014; Kuzmanoski et al., 2015; Alammar et al., 2016; Sharma et al., 2017a). Eventually, during the synthesis, various types of structure-controlling agent(s) and volatile organic compounds are used for tuning the size, morphology, crystal phase of prepared nanophosphors so that desired materials can be synthesized (Gai et al., 2014; Sharma et al., 2017a). However, volatile organic compounds have hazardous impacts on flora and fauna. To overcome these issues, ionic liquid (IL) assisted methods have been developed as they have high thermal and chemical stability, less volatility, and tunable physicochemical properties, which make them superior to conventional organic solvents (Rogers and Seddon, 2003; Wilkes, 2004; Plechkova and Seddon, 2008; Cybinska et al., 2016). Therefore, ILs are also known as “green” and “designer” solvents (Rogers and Seddon, 2003; Plechkova and Seddon, 2008; Duan et al., 2014). Thus, ILs can also be employed as solvents, reaction precursors, and structure-directing agents in the synthesis of nanomaterials (Liu et al., 2010; Li et al., 2011a; Kundu et al., 2012; Cybińska et al., 2016; Ghosh and Mudring, 2016; Sharma et al., 2020a). In this review article, we have provided a brief introduction of IL and its applications and role in various fields including nanomaterials synthesis and designing, especially for Ln^3+^-doped nanophosphors. Thereafter, the origin of various photophysical processes of Ln^3+^-doped nanophosphors such as energy transfer, upconversion, and downconversion and the factors influencing these photophysical processes of Ln^3+^ ions are discussed. In addition, a brief overview of Ln^3+^-IL complexes is presented.

Finally, the applications of Ln^3+^-doped nanophosphors in white light emitting materials, optical sensors, solar cells, and imaging purposes are discussed.

## Fundamentals of Lanthanide Ions and Origin of Their Spectroscopic Features

There are seventeen elements in the periodic tables [including Sc (21), Y (39), and La (57)–Lu (71)] that are considered as rare-earth elements and commonly exhibit +3 (III) oxidation states (Gai et al., 2014; Sharma et al., 2017a). In addition, other elements may also exhibit +4 and +2 oxidation states such as Sm^2+^, Ce^4+^, Eu^2+^, Tb^4+^, and Yb^2+^ ions (Su et al., 2002; Anghel et al., 2017; Runowski et al., 2020a; Li and Zhang, 2020). The electronic configuration of the lanthanide (Ln^3+^) series, particularly in the +3 state, is represented as [Xe] 4*f* ^n^. Commonly, intraconfigurational *f-f* electronic transitions are occurring (Sharma et al., 2017a). One of the interesting aspects of the RE^3+^ ions is the presence of numerous metastable energy levels between the ground and the excited state for electronic transitions to occur. These energy levels can be calculated by 14!/(14-n)!n!, where n is the number of electrons in *f*-orbitals (Sharma et al., 2017a). The optoelectronic features of the RE^3+^ ion(s)-doped nanocrystals generally originate due to size-independent (quantum-mechanically, not quantum-confined) and parity-forbidden *f-f* electronic transitions except Ce^3+^ ions, as the emission of Ce^3+^ ion is a consequence of spin-allowed *f-d* electronic transition (Sharma et al., 2017a; Sharma et al., 2020a). The penultimate orbitals of the lanthanide series are highly shielded by filled *5s* ^*2*^ *5p* ^*6*^ orbitals (Guo et al., 2010; Gai et al., 2014; Sharma et al., 2017a). Therefore, the crystal field slightly influences the electronic transition of the RE^3+^ ions leading to the narrow excitation and emission bands of RE^3+^ ions doped in nanomaterials, which is contrary to the electronic transitions of the d-block elements (transition elements) (Gai et al., 2014; Sharma et al., 2017a). Normally, in RE^3+^ ions, very weak oscillator strength is found with an absorption coefficient of less than 10 M^−1^cm^−1^ (Sharma et al., 2017a). On the other hand, La^3+^ and Lu^3+^ do not show luminescence features due to either empty or filled *f-*orbitals. However, the photophysics of the RE^3+^-doped nanocrystals can be tuned by changing the crystal phase, shape, lattice strain, core-shell structures, and nature of host materials (Gai et al., 2014; Sharma et al., 2017a).

### Photophysics of Ln-Doped Nanomaterials

The photophysical processes of the rare-earth-doped nanomaterials are varied according to the incorporation of dopant ions. Due to the presence of various metastable energy levels in the RE^3+^ ions, the possibility of numerous emission lines increases and can be found through Dieke’s diagram (Sharma et al., 2017a). As a result, excitation and emission energies are varied with RE^3+^ ions (Ghosh et al., 2011; Zhang et al., 2012; Lorbeer and Mudring, 2014; Goldschmidt and Fischer, 2015; Ghosh and Mudring, 2016).

**FIGURE 1:**
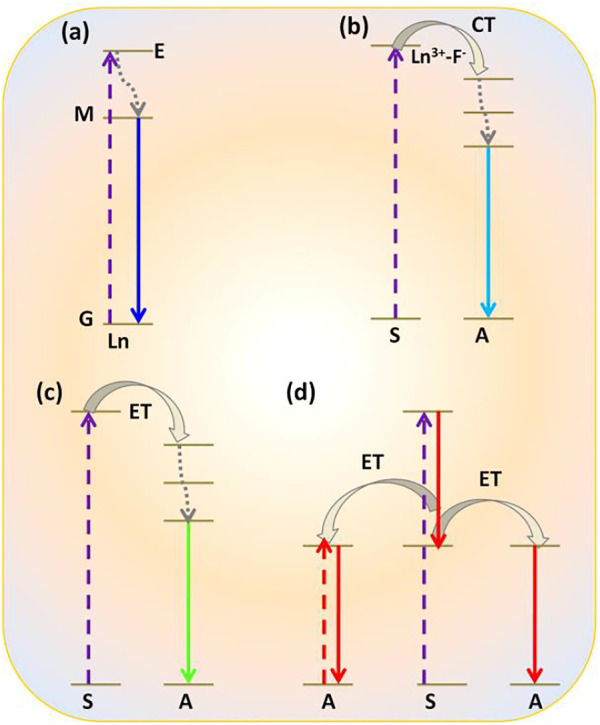
Photophysical processes shown by RE^3+^-doped nanomaterials: **(A)** downshifting; **(B)** charge transfer (CT); **(C)** energy transfer (ET); **(D)** quantum cutting downconversion.

**FIGURE 2:**
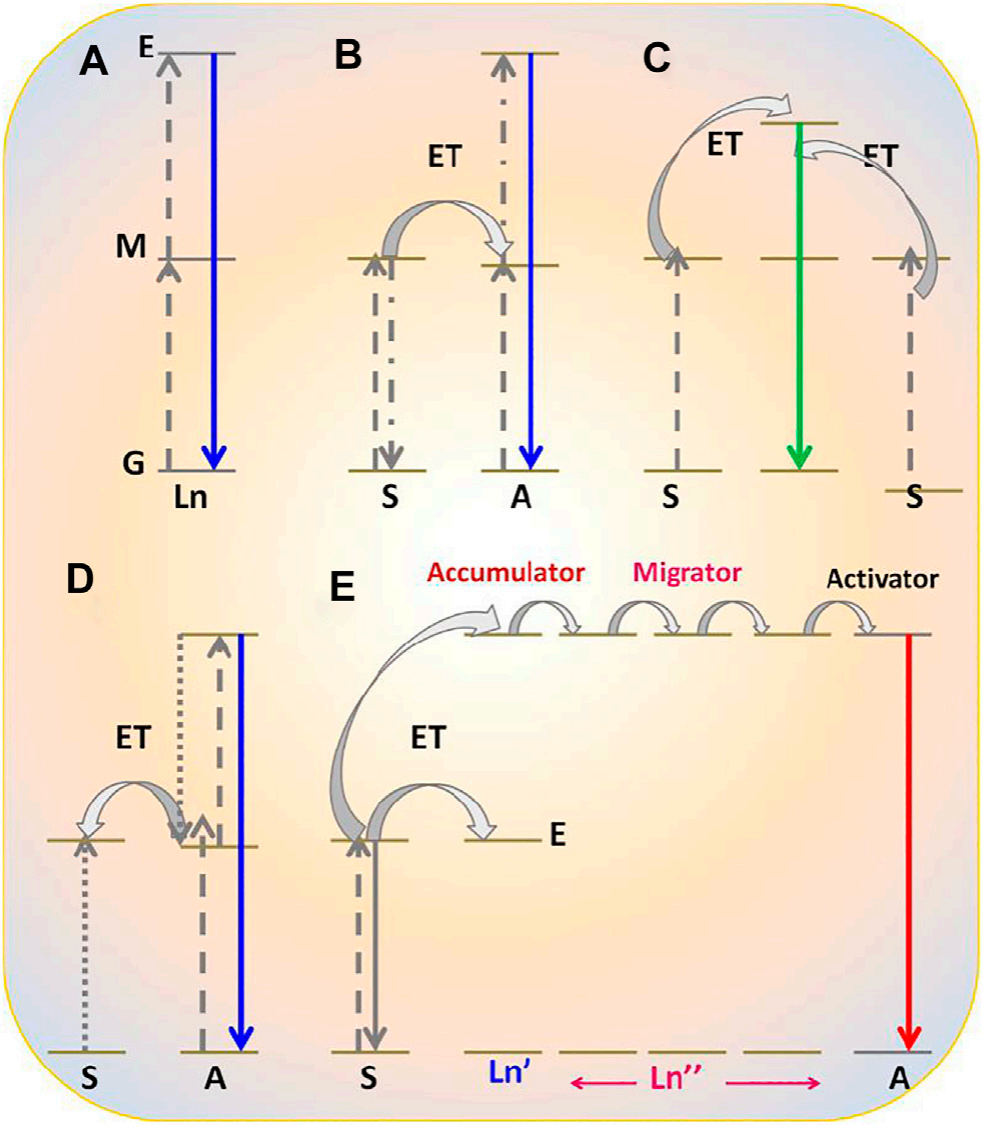
Upconversion photophysical processes shown by RE^3+^-doped nanomaterials: **(A)** excited state absorption; **(B)** energy transfer upconversion; **(C)** cooperative energy transfer upconversion; **(D)** photon avalanche; **(E)** energy migration-mediated upconversion.

Commonly, photophysical processes exhibited by RE^3+^ ions can be classified into five categories: direct excitation or downshifting, charge transfer, energy transfer, and quantum cutting downconversion and upconversion processes (see Figures 1, 2). In this section, all these processes are described in detail.

#### (i) Downshifting

In this process, the electron of RE^3+^ ions is excited in the presence of high energy, i.e., by UV radiation to the uppermost level of the excited state. From this level, it comes to a lower excited level *via* non-radiative emission and then finally by radiative emission; then, it returns again to the ground state. It is a process that happens regularly (Figure 1A) (Sharma et al., 2017a).

#### (ii) Charge Transfer

Charge transfer is associated with the transition from 2p orbital of oxide (O^2-^) or fluoride (F^−^) to the excited level of RE^3+^ ions, for example, incomplete 4f orbital of Eu^3+^ ions (Ghosh et al., 2011; Alammar et al., 2016; Ghosh and Mudring, 2016). Thereafter, the electron from the excited level comes to the lower excited level through the non-radiative relaxation process. Then, it reaches the ground state by radiative emission. Due to the forbidden characteristics (by Laporte selection rules) of the 4f-4f transitions in lanthanide ions (Ln^3+^), their direct excitation is ineffective, and the absorption coefficient is usually very small. Such a type of photophysical process is generally noticed in red-emitting Eu^3+^ ion-doped oxides and fluorides materials (Ghosh et al., 2011; Alammar et al., 2016; Ghosh and Mudring, 2016). Upon excitation at 254 nm, the electron is transferred from O^2-^ to Eu^3+^ ions. Due to the large bandgap and low vibrational energy of the host matrix, as in the case of Eu^3+^-doped REF_3_/MREF_4_ nanoparticles, Eu^3+^-F^−^ bond is extensively ionic in nature compared to Eu^3+^-O^2-^ bond (Sharma et al., 2017a). Consequently, high-energy radiation (<180 nm) is required for charge transfer transition from F^−^ to Eu^3+^ ion, leading to red emission by Eu^3+^ ions (Ghosh and Patra, 2008; Ghosh et al., 2011; Ghosh and Mudring, 2016).

#### (iii) Energy Transfer

It is already illustrated that RE^3+^ ions generally have a low absorption coefficient compared to transition metal ions (Gai et al., 2014; Sharma et al., 2017a). Therefore, direct transition in 4*f* ^*n*^ energy levels is restricted in most cases and inefficient if it occurs. To overcome this problem, sensitizers (S) with larger absorption cross-sections are often used to absorb the irradiating energy, which again can be easily transferred to high-energy levels of the activators (Gai et al., 2014; Sharma et al., 2017a). These sensitizers are often excited using either high-energy photons of ultraviolet range or infrared photons. For instance, Ce^3+^ ions and Yb^3+^ ions (absorption coefficient of Yb^3+^ ∼ 9.11 × 10^−21^ cm^−2^) are considered as efficient sensitizers (S) (Dong et al., 2015). After absorbing the irradiating photons from the source by sensitizer, it is transferred to the highest excited level of nearby RE^3+^ ions *via* the energy transfer process. Subsequently, emission of either higher or lower energy of photons takes place from the RE^3+^ ions known as an activator (A). The most common examples of such photophysical processes are the Ce^3+^ and Tb^3+^ ions co-doped nanoparticles and Yb^3+^ and Ho^3+^/Er^3+^/Tm^3+^ co-doped nanoparticles (Zharkouskaya et al., 2008; Zhang and Chen, 2015; Liu et al., 2018; Zhao et al., 2018). Upon exciting Ce^3+^ ions in the UV region of photons, excited electrons are promoted from *4f* to empty orbitals of *5d* followed by radiative emission from ^2^D to ^2^F_5/2,7/2_ lower energy level of Ce^3+^ (Ghosh et al., 2010). On the other hand, when Tb^3+^ is doped along with Ce^3+^, due to the degree of overlapping of excitation region of Tb^3+^ ions and emission region of Ce^3+^ ions, efficient energy transfer takes place from Ce^3+^ to Tb^3+^ ions (Zharkouskaya et al., 2008; Zhang et al., 2012; Zhang and Chen, 2015). Despite the occurrence of blue emission, green emission is observed by Tb^3+^ ions that can be attributed to transitions from ^5^D to underlying ^5^F_J_ levels of Tb^3+^ ions. (Ghosh et al., 2010). Another example of the ET process is Yb^3+^ and Er^3+^ ions co-doped nanoparticles, where Er^3+^ ions have ladder-like energy levels. So, when exciting the Yb^3+^ ions with low-energy photons from ^2^F_7/2_-^2^F_5/2_ where energy corresponds to the NIR region (980 nm), energy is successively promoted to higher energy levels of Er^3+^ ions followed by radiative emission in visible region photon (Ghosh et al., 2008a; Dong et al., 2015). This radiative emission is called upconversion emission. In addition to green emission by Er^3+^ ions, Ghosh et al. have reported the red and green emission in Yb^3+^ and Er^3+^- co-doped LaPO_4_ nanoparticles. The emission of red light centered at 670 nm is attributed to the radiative relaxation of Er^3+^ ions from ^4^F_9/2_ to the ground state (^4^I_5/2_) (Ghosh et al., 2008a).

#### (iv) Quantum Cutting Downconversion

Quantum cutting downconversion is basically concerned with converting high-energy single photon to more than one photon of lower energy. In this case, sensitizer generally has one or more metastable states (Figure 1D) (Ghosh and Mudring, 2016; Lorbeer and Mudring, 2014; Ghosh et al., 2011; Lorbeer et al., 2010; Wegh et al., 1999). During the process, the sensitizer gets excited after absorbing the high-energy UV photon. Two situations arise: first, as the excited electron is relaxed to a metastable state, released energy is transferred to the highest excited level of the activator via the ET process. Thereafter, the electron comes down to the lower excited level of the activator through the non-radiative process. Finally, it is then relaxed to the ground state via the radiative emission of one lower energy photon (Figure 1D). The second situation arises when released energy is simultaneously absorbed by another activator and gets excited to its higher energy level. Thereafter, it is relaxed to its ground state, followed by the emission of a second lower energy photon. In this way, finally, one can get a maximum of two lower energy photons after absorbing a single high-energy photon. Such photophysical processes often occur in lanthanide fluoride like GdF_3_ or alkali and alkaline earth fluorides nanomaterials such as MGdF_4_/M’GdF_5_ (M = Li^+^, Na^+^, K^+^, and M’ = Ca^2+^, Ba^2+^, Sr^2+^) doped with Eu^3+^ ions nanocrystals. Here, Gd^3+^ is often used as sensitizer and Eu^3+^ ion as an activator (Ghosh and Mudring, 2016; Lorbeer and Mudring, 2014; Ghosh et al., 2011; Lorbeer et al., 2010; Wegh et al., 1999). For instance, Eu^3+^-doped NaGdF_4_ nanomaterials absorb the high-energy UV light and emit visible light in the red region (Ghosh and Mudring, 2016; Ghosh et al., 2011; Chouryal et al., 2021). However, when Er^3+^ and Tb^3+^ are doped in the NaGdF_4_ host matrix, green-emitting quantum cutting downconversion is observed (Figure 1D) (Lorbeer and Mudring, 2014).

#### (v) Upconversion

The upconversion process is the opposite of a downconversion process. In this process, an anti-Stokes shift is observed in which low-energy excitation photon of infrared region light is converted into the high-energy emission photon of visible light. Contrary to second-harmonic generation, upconversion takes place via the available intermediate energy levels (Figures 2A–E) (Gai et al., 2014; Dong et al., 2015; Sharma et al., 2017a).

Therefore, it is also called “Addition de Photons par Transfert d'Énergie” (APTE). In the mid-1960s, this process was independently discovered by F. Auzel and Ovsyankin and Feofilov (Auzel, 2004; Feofilov and Ovsyankin, 1967). The upconversion process can be further divided into five groups: excited state absorption, energy transfer upconversion, cooperative energy transfer upconversion, and energy migratory-mediated upconversion (Figures2A–E) (Feofilov and Ovsyankin, 1967; Auzel, 2004; Gai et al., 2014; Dong et al., 2015).

##### (A) Excited State Absorption

The excited state absorption process is an upconversion process in which the RE^3+^ ion is excited by two pumped photons with lower energy. As a result, a high-energy photon is emitted. Normally, this process needs RE^3+^ ions with a larger absorption cross-section, high pump power density, and low dopant concentration (preferably 1%). As there is no sensitizer used, this process is normally less efficient (Figure 2A). For example, when Er^3+^ ions are irradiated with infrared photons, different intermediate energy levels between ground and excited states get populated. Moreover, when they are radiatively relaxed to the ground state, excited state upconversion is observed (Zou and Izumitani, 1993; Ghosh et al., 2008b).

##### (B) Energy Transfer Upconversion

Energy transfer upconversion is a highly efficient and widely studied upconversion process, where sensitizer is co-doped with activator. As the activator has numerous, very close ladder-like metastable states, during this process, the sensitizer is excited by low-energy photons and resultant energy is transferred to the activator (Gai et al., 2014; Dong et al., 2015; Sharma et al., 2017a). After absorbing the released energy through energy transfer process, the activator reaches the excited level via intermediate energy levels, which are situated between the ground and excited states. Thereafter, different energy of photons in the visible region is radiatively emitted after relaxing from the subsequent energy levels. Herein, Yb^3+^ is used as a sensitizer with a larger absorption cross-section, and Tm^3+^, Er^3+^, and Ho^3+^ are often used as activators to be co-doped with sensitizers (Gai et al., 2014; Dong et al., 2015; Sharma et al., 2017a).

##### (C) Cooperative Energy Transfer Upconversion

Eventually, it is a less studied process and different from the previously mentioned two upconversion processes. This process usually occurs between pairs of Yb^3+^ ions. In this process, the absorbed energy by the sensitizer is transferred to a quasi-virtual state from which radiative transition occurs to the ground state (Feofilov and Ovsyankin, 1967). However, it is also a less efficient process than the energy transfer upconversion process.

##### (D) Photon Avalanche Upconversion

Photon avalanche (PA) is a type of upconversion process that is seldomly noticed. This process was first discovered by Chivian et al. in 1979 for illustrating the quantum counter behavior of Pr^3+^-doped in LaCl_3_ and LaBr_3_ (Chivian et al., 1979). The prerequisite condition for this process is that laser pump radiation should exceed the critical intensity. The mechanism of PA can be seen in Figure 2D. Initially, a normal upconversion process takes place. As the relaxation from excited level to metastable state occurs, energy is transferred to another neighbor ion of the same species for exciting it from its ground state to metastable state, followed by the population of metastable state again. In this way, the metastable state is always populated, and this process keeps continuing (Chivian et al., 1979).

##### (E) Energy Migratory-Mediated Upconversion

This novel upconversion process (Figure 2E) was discovered recently and is known as EMU (energy migration-mediated upconversion). It was observed in core-shell nanostructure NaGdF_4_:Yb^3+^, Tm^3+^@NaGdF_4_:RE^3+^ (RE^3+^ = Tb, Eu, Dy, and Sm) (Wang et al., 2011). The noticeable feature of this process is that four types of rare-earth ions are utilized for serving different functions such as sensitizer, accumulator, migratory, and activator. Yb^3+^ ion as sensitizer absorbs the pumping photons and subsequently transfers them to an ion situated in its vicinity, called accumulator ion (Tm^3+^), to excite it at the excited level. Thereafter, the photon is transferred from the high-energy levels of the accumulator (Tm^3+^) to a migratory ion (Gd^3+^).^48^ This energy migration process keeps occurring in the materials until energy is transferred to the activator ion. Finally, the activator is relaxed to its ground state via radiative emission of the characteristic visible region of the photon.

### Impact of Host Materials

Judicious selection of host material is very important for tuning the photophysics of RE^3+^ dopant ions (Sharma et al., 2017a). Numerous types of host materials have been explored to date, including oxides, phosphates, fluorides, vanadate, and borate (Sun and Zheng, 2010; Tian et al., 2012; Tian et al., 2014; Kuzmanoski et al., 2015; Alammar et al., 2016; Cybinska et al., 2016; Muthulakshmi et al., 2020; Chouryal et al., 2021). Important points have to be considered before selecting the host material like thermal and chemical stability, low phonon energy, high refractive index, and large bandgap (Gai et al., 2014; Sharma et al., 2017a). Considering all aspects, fluoride-based host materials are considered to be a better host matrix for doping of the RE^3+^ ions compared to the other host materials (Gai et al., 2014; Sharma et al., 2017a). Another important feature of the fluoride-based host matrices is that they occur in different polymorphs, can be tuned by tuning temperature, reaction precursors, dopant concentration, and size (Gai et al., 2014; Sharma et al., 2017a). Besides fluorides, phosphate- and oxides-based materials are also used (Bühler and Feldmann, 2007; Zharkouskaya et al., 2008; Kowsari and Faraghi, 2010; Tian et al., 2012; Zou et al., 2013; Tian et al., 2014; Alammar et al., 2016; Liu et al., 2020). However, the fluorides-based nanomaterials have thermal stability issues, particularly in their applications at high temperatures. It has been noticed that upon heating the fluorides-based nanomaterials at high temperatures, sometimes oxyfluorides are formed (Knudson, 1954). In order to tackle these issues, oxides-, phosphates-, borate-, vanadates-, and molybdates-based nanomaterials have gained noticeable attention. In addition, the morphology of the host material also influences the optical properties of the doped rare-earth ions. Several methodologies have been developed so far to prepare different host materials (Gai et al., 2014; Sharma et al., 2017a). Herein, we have focused mainly on the task-specific IL-assisted synthesis of RE-doped nanomaterials. Ionic liquids play a very important role in designing host materials, which will be discussed in this chapter.

## Lanthanide-based Nanomaterials *via* Ionic Liquids

### What Is Ionic Liquid: Past and Present Scenario?

**FIGURE 3:**
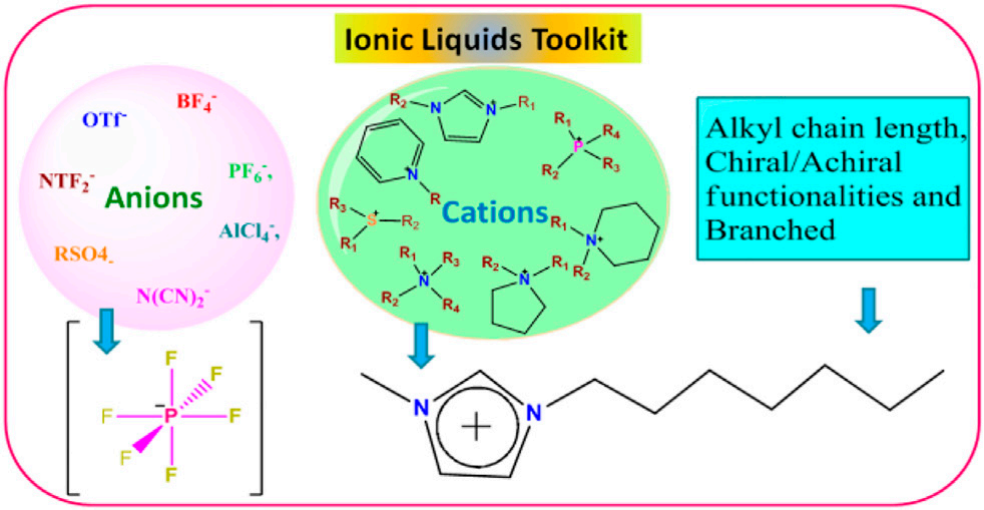
Ionic liquids (ILs) toolkit consists of cations and anions.


ILs have become an important part of chemistry, materials science, and electrochemistry. Recently, they have got tremendous attention for several applications due to their tunable properties. Ionic liquids (ILs) are organic salts that have a melting point less than 100°C in ambient condition and are comprised of cation and anion (Rogers and Seddon, 2003; Wilkes, 2004; Plechkova and Seddon, 2008; Roth et al., 2012; Hallett and Welton, 2011; Armand et al., 2009; Krossing et al., 2006). Therefore, many combinations (maximum 10^18^) of cation and anion are possible, leading to the formation of a variety of ionic liquids (Rogers and Seddon, 2003; Wilkes, 2004; Plechkova and Seddon, 2008; Roth et al., 2012; Hallett and Welton, 2011; Armand et al., 2009; Krossing et al., 2006). Besides this, different types of IL cations can also be produced by substituting with the desired alkyl chain length on the fundamental cations, such as imidazolium, pyridium [C_5_H_5_NR]^+^, pyrolidinium [C_5_H_10_NR_2_]^+^, phosphonium [PR_4_]^+^, sulphonium [SR_3_]^+^, and alkylammonium [NR_1_R_2_R_3_R_4_]^+^ (here, R_1_ = -H, alkyl and R_2,3,4_ = alkyl groups). In addition, by changing the anions such as X^−^ (Cl^−^, Br^−^), BF_4_^−^, PF_6_^−^, OTf^−^, RSO_4_^-^, and OH^−^, one can get ILs with different properties (see Figure 3) (Rogers and Seddon, 2003; Wilkes, 2004; Krossing et al., 2006; Plechkova and Seddon, 2008; Armand et al., 2009; Hallett and Welton, 2011; Roth et al., 2012). As a result of these incredible characteristics of the ILs, chemical and physical properties can be feasibly tuned according to the necessity of reaction conditions; for these reasons, ionic liquids are often called “*designer*” solvents (Plechkova and Seddon, 2008; Roth et al., 2012). ILs came into the picture a century ago, when ethylammonium nitrate salt was recognized as molten salt (Plechkova and Seddon, 2008; Roth et al., 2012). The application of ionic liquids was assured when pyridinium salt was used to dissolve cellulose at 100°C (Plechkova and Seddon, 2008; Hallett and Welton, 2011). Then, for reprocessing the nuclear fuel, chloroaluminates- (AlCl_4_^−^-) based ionic liquid of low melting temperature was used (Plechkova and Seddon, 2008; Armand et al., 2009; Hallett and Welton, 2011). The major drawback of these ionic liquids was their high sensitivity to atmospheric moisture and protonic impurities (Plechkova and Seddon, 2008; Armand et al., 2009; Hallett and Welton, 2011; Roth et al., 2012). For some time, other low melting ionic liquids were explored especially using imidazolium cations, and the sensitivity of ILs towards moisture and reaction medium (acidic and basic medium) problem was sorted out by applying plasticizing anions, for instance, bis(trifluoromethylsulfonyl)amide (NTf^−^) (Roth et al., 2012; Hallett and Welton, 2011). In this anion, CF_3_SO^−^ groups are a strong electron-withdrawing group bound to the N atom, leading to the formation of flexible S-N-S bonds. NTf^−^ anion containing ILs have a low melting point; for instance, −15°C is reported for the 1-ethyl-3-methylimidazolium-based IL (Roth et al., 2012). Another example of the influence of anions on the physical and chemical characteristics of ILs is noticed when BF_4_^−^ and PF_6_^−^ anions were used with imidazolium cations. The melting point of the obtained ionic liquids is substantially altered and lowered down even less than room temperature compared to the halides containing ILs (shown in Table 1) (Rogers and Seddon, 2003; Armand et al., 2009). As a result, these characteristics of ILs render them superior to other conventional solvents.

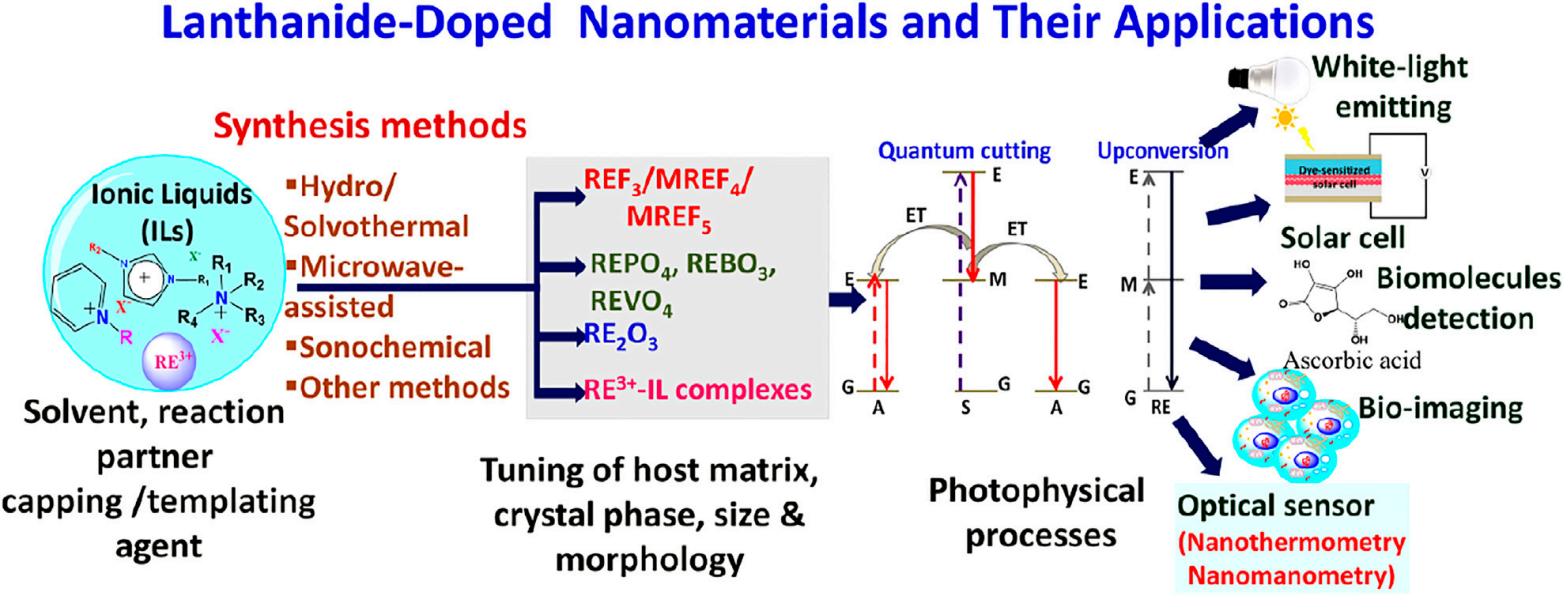


**TABLE 1:** Melting point, viscosity, and electrical conductivity of ionic liquids (Faridbod et al., 2011; Wang et al., 2007; Shukla and Sah, 2013).

Ionic liquids	Melting point (T_m_, K)	Viscosity (*η*, cP)	Electrical conductivity (S/m)
[C_4_mim][Br]	349.2	Solid	—
[C_4_mim][BF_4_]	192.2	219	0.35
[C_4_mim][PF_6_]	277	450	0.14
[C_4_mim][Tf_2_N]	248	69	0.40

Though ionic liquids are extensively ionic, they still have a low melting point, even less than 0°C. The low melting point of ILs was determined based on theoretical calculations. It was found that due to the large size of constituent ions (cation and anion) of ILs, generally high conformational flexibility leads to fewer lattice enthalpies and high entropy value, favoring the low melting point (Krossing et al., 2006). In addition, Krossing et al. have used the Born–Fajans–Haber cycles to estimate the Gibbs free energy (∆_fus_G^T^) of fusion (IL(s) → IL(l)) of ILs that depends on the lattice (IL(s) → IL(g)) Gibbs energy (∆_latt_G^T^) and solvation (IL(g) → IL(l)) Gibbs energies (∆_solv_G^T^). The value of ∆_fus_G is negative for ILs, meaning that ILs exist in a liquid state (Krossing et al., 2006).

### How Are Ionic Liquids Better Than Conventional Liquids?

In the past, conventional organic solvents were extensively used for synthesizing, especially nanomaterials. The major problems concerned to those solvents were their high volatility and low decomposition temperature. In addition, thermal decomposition, particularly at high temperatures, leads to the release of hazardous gases, which cause serious environmental problems (Rogers and Seddon, 2003; Plechkova and Seddon, 2008; Armand et al., 2009). Therefore, all these issues of conventional solvents always motivated the scientific groups to explore new environmentally benign solvents. As a result, with the persistent effort of many scientists, ionic liquids were explored and continuously developed in the scientific domain. Ionic liquids have several attractive features, especially tunable properties. Just by changing the combination of cation and anion, new properties such as high thermal stability (even though >250°C), large electrochemical window spanning 6V, negligible vapor pressure, high liquidus range can be obtained (Rogers and Seddon, 2003; Plechkova and Seddon, 2008; Armand et al., 2009). Due to these interesting physicochemical properties, ILs have several applications in industries and organic synthesis (Rogers and Seddon, 2003; Plechkova and Seddon, 2008; Armand et al., 2009; Hallett and Welton, 2011). In addition, they are used not only as solvents but also as reaction partners for the source of desired ions. In this way, ionic liquids can be considered potential solvents for several applications, including nanomaterial design discussed in the next section.

**Characterization of ionic liquids**: the purity of ILs is a prerequisite for numerous applications, including nanomaterials synthesis. Several instrumental techniques are employed to check the purity of ILs, for example, to check whether the as-prepared IL is free from any kind of impurities such as unreactive reaction precursor, side products, and, most importantly, moisture. The most common characterization techniques are nuclear magnetic resonance (NMR), FTIR to determine the nature of cations and anions present in the IL, and characteristic vibrational frequencies of different groups like alkyl chain length, imidazolium ring, and hydroxyl group (in case of moisture) qualitatively (Cybinska et al., 2016; Sharma et al., 2017b). The moisture content in the as-synthesized ILs can be determined using the Karl–Fischer titration method. (Widegren et al., 2005).

### Important Contribution of IL(s) in Nanomaterials Engineering

**FIGURE 4:**
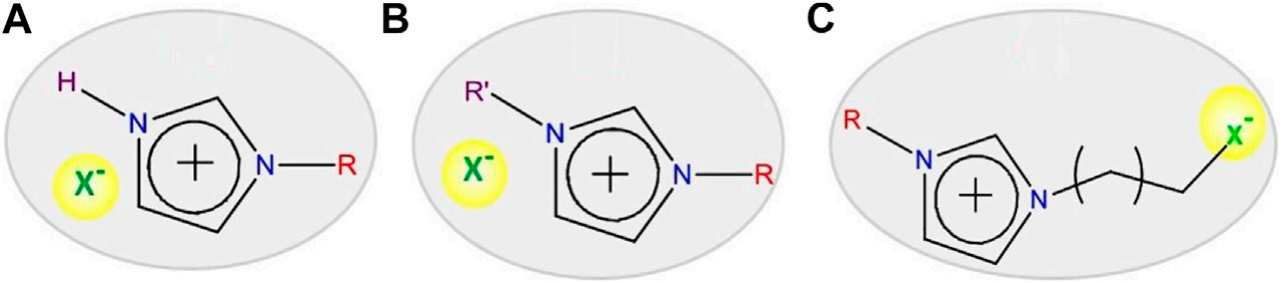
Task-specific ionic liquids for particular applications: **(A)** protic, **(B)** aprotic, and **(C)** zwitterionic.

ILs are important as a widespread tunable class of solvents that are used in several fields because of their unique properties. The application of ILs is a function of their composition. Therefore, judicious selection of cation-anion combinations of ILs is necessary to tune the physicochemical properties of ILs. Normally, ILs can be classified based on their applications in various fields into three groups: protic, aprotic, and zwitterionic types (shown in Figure 4) (Armand et al., 2009).

**FIGURE 5:**
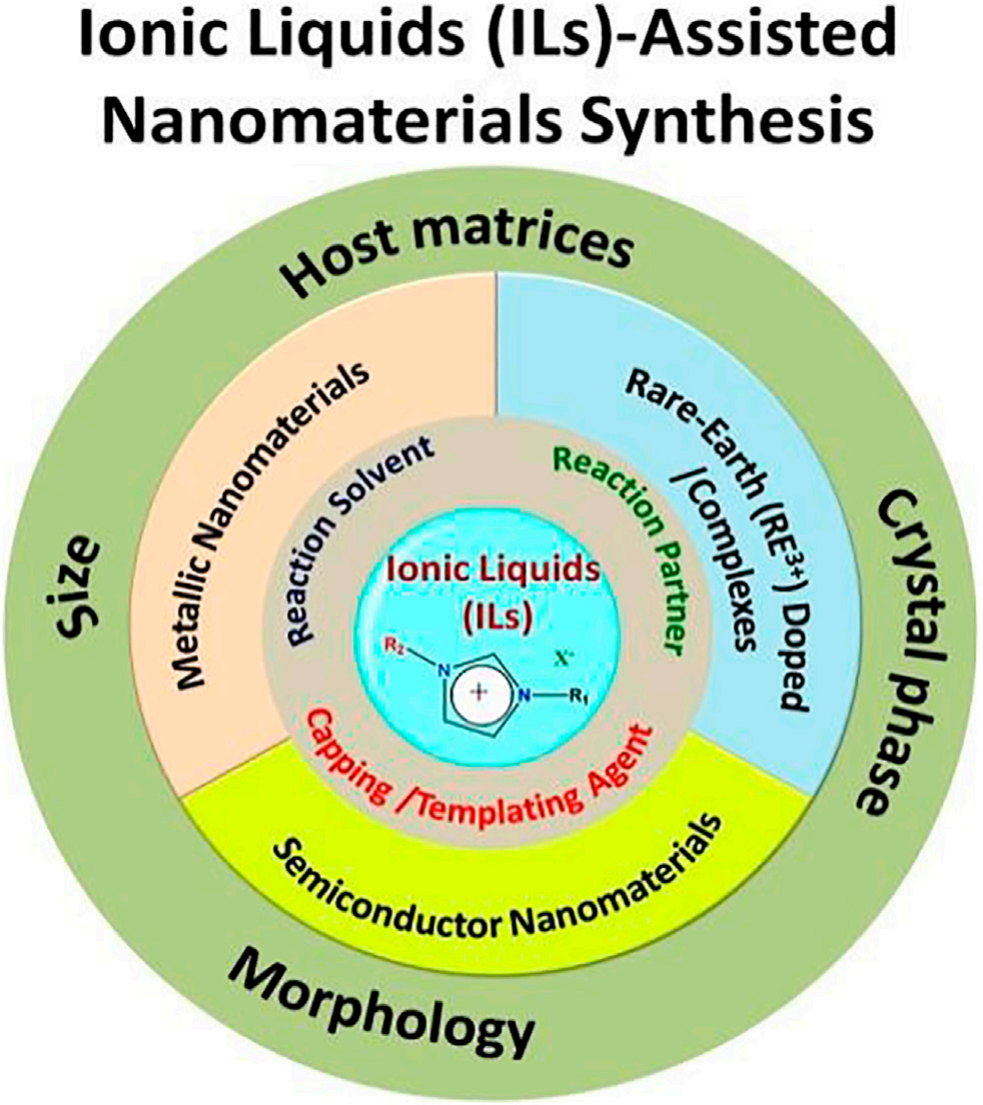
The role of ionic liquids in the synthesis of various classes of nanomaterials.

Protic ILs are widely used in fuel cells and aprotic ILs in Li-ion batteries and supercapacitor applications. On the other hand, zwitterionic-based ILs are used to prepare IL-based membranes (Armand et al., 2009). In addition, other important applications of ILs are catalysis of organic reactions, energy resources, conversion of CO_2_ into useful organic compounds for industrial applications, reaction medium to perform the reactions, extraction of rare-earth metal compounds from their minerals, separation of toxic elements, and reaction partner for synthesizing the inorganic-based nanomaterials (Plechkova and Seddon, 2008; Duan et al., 2014; Hallett and Welton, 2011; Armand et al., 2009; Guterman et al., 2018; Azov et al., 2018; Zhang et al., 2014; Fabregat-Santiago et al., 2007; Visser et al., 2002). Despite these applications, only recently did ILs receive considerable attention in the synthesis of nanomaterials (Figure 5). During nanomaterial synthesis, ILs are used not only as solvents but also as reaction partners and capping/templating agents and as nanoreactors due to the presence of tunable alkyl chain length (Duan et al., 2014).

Earlier, ILs have been used to catalyze organic and organometallic synthesis (Visser et al., 2002; Hallett and Welton, 2011). On the other hand, Dupont's group have prepared uniform Ir nanoparticles using [C_4_mim][PF_6_] IL as reaction media (Dupont et al., 2002). In 2004, Taubert synthesized CuCl nanoplatelets using the IL-crystal as precursor (Taubert, 2004). The term “all-in-one” is used to describe ILs because these are usually used as solvent, precusors, and stabilizing agents in the preparation of inorganic materials (Richter et al., 2013; Duan et al., 2014). In the beginning, ILs were employed to not only synthesize the metallic nanoparticles but also to synthesize semiconducting nanomaterials (Richter et al., 2013; Duan et al., 2014). In the course of the reaction, ILs were applied as reaction partners and reaction media. For instance, anions parts of IL such as [BF_4_^−^, PF_6_^−^, bromide (Br^−^), iodide (I^−^)], [H_2_PO_4_]^-^, and [SeO_2_(OCH_3_)]^−^can be used as a source of halides, phosphate ion (PO_4_^3−^), and selenide (Se^2−^) ion, respectively (Lorbeer and Mudring, 2013a; Richter et al., 2013; Duan et al., 2014; Cybinska et al., 2016). [C_16_mim][Br] and [C_4_mim][I] ILs are applied to make BiOBr and BiOI nanoparticles, respectively (Xia et al., 2011a; Xia et al., 2011b). On the other hand, using the [C_4_mim][SeO_2_(OCH_3_)], several selenide-containing nanoparticles have been prepared, such as ZnSe and Cu_2_Se (Liu et al., 2010; Duan et al., 2011; Liu et al., 2011). Moreover, ILs can control crystal phase and morphology and stabilize the nanoparticles (Kowsari and Faraghi, 2010; Ghosh and Mudring, 2016). Furthermore, ILs can also be used to reduce the metal ion for preparing the metallic cluster (Liu et al., 2010; Xia et al., 2011a; Xia et al., 2011b; Duan et al., 2011; Liu et al., 2011; Richter et al., 2013). For stabilizing the nanoparticles, various modes of interaction generally take place; for example, IL can be attached to the surface of nanoparticles by H bonding through acidic proton and aromatic π-system and via interaction with anions (Pensado and Pádua, 2011; Richter et al., 2013).

### Preparative Methods for Lanthanide Ion(s)-Doped Nanomaterials Using IL

Numerous preparative methods can be used to synthesize RE-based nanomaterials. However, during the last decade, IL-assisted synthesis approaches became a novel way to prepare RE-based nanomaterials. IL-based synthesis approaches not only aid in the formation of desired products but also control the size and morphology and assist in the functionalization of as-prepared products. Some of the state-of-the-art synthesis techniques, such as IL-assisted hydrothermal/solvothermal, microwave-assisted and sonochemical techniques, are discussed in this review article (shown in Table 2).

**TABLE 2:** Nanomaterials, synthesis methods, ionic liquids, role of ILs, and morphology of nanoparticles.

Nanomaterials	Synthesis method	Ionic liquid(s)	Role of ionic liquids	Morphology
YPO_4_:Eu^3+^	Hydrothermal	[Choline][H_2_PO_4_]	Reaction partner, solvent	Nanopowders (Cybińska et al., 2016)
SmVO_4_	Hydrothermal	[C_4_mim][Br]	Solvent capping agent	Nanosheets (Sun and Zheng, 2010)
BaF_2_:Eu^3+^	Solvothermal	[C_2_mim][Br]	Capping agent	Cubical (Sharma et al., 2019)
BaF_2_:Ce^3+^	Solvothermal	[C_4_mim][BF_4_]	Reaction partner solvent capping agent	Flakes like (Sharma et al., 2020a)
YF_3_:Eu^3+^	Hydrothermal	[DADMA][BF_4_]	Reaction partner capping agent	Nanorods (Wang et al., 2015a)
LaF_3_:Eu^3+^				
GdF_3_:Eu^3+^				
BaF_2_:Ce^3+^/Tb^3+^	Solvothermal	[C_2_mim][Br]	Capping agent	Cubical (Chouryal et al., 2020)
YF_3_:Ln^3+^ (Ln = Eu, Tb, Ce, Dy)	Hydrothermal	[C_8_mim][PF_6_]	Reaction partner, capping agent	Nanorhombi (Li et al., 2011b)
LaF_3_:Ce^3+^,Tb^3+^	Hydrothermal	[C_4_mim][BF_4_]	Reaction partner solvent	Nanodiskette (Guo et al., 2010)
YF_3_	Hydrothermal	[C_4_mim][BF_4_][C_4_mim][PF_6_]	Reaction partner solvent capping agent	Spherical
				Spindle
				Nanorods (Zhong et al., 2009)
Ca_5_(PO_4_)_3_Cl:Ce^3+^,Tb^3+^	Hydrothermal	[C_8_mim][Cl]	Reaction partner solvent capping agent	Sheaves microrods (Zou et al., 2013)
BiOBr:Er^3+^	Solvothermal	[C_16_mim][Br]	Reaction partner capping agent	Microspheres (Xia et al., 2016)
Y_7_O_6_F_9_: Yb^3+^-Tm^3+^	Hydrothermal	[C_4_mim][BF_4_]	Reaction partner solvent capping agent	Petal shaped microsphere (Zhao et al., 2018)
Y_2_O_3_	Solvothermal	[C_4_mim][Br]	Capping agent	Square-shaped Nanoplates (Wang et al., 2015b)
Na_3_Y_1-x_(PO_4_)_2_: xTb^3+^	Hydrothermal	[Choline][H_2_PO_4_]	Reaction partner	Spindle-shaped particles (Liu et al., 2020)
LuF_3_:Ln^3+^(Ln = Eu, Tb, Dy)	Solvothermal	[C_8_mim][PF_6_]	Reaction partner capping agent	Rhombic spindle-shaped (Liu et al., 2014a)
NaGdF_4_:Eu^3+^	Solvothermal	[C_2_mim][Br]	Capping agent	Nanorods (Ghosh and Mudring, 2016)
		[C_2_dmim][Br]		
		[C_2_mim][Cl]		
		[C_4_mim][Br]		
		[C_6_mim][Br]		
		[C_8_mim]]Br]		
		[C_10_mim][Br]		
		[Me_4_N][Br]		
LaF_3_:Tb^3+^	Hydrothermal	[C_4_mim][BF_4_]	Reaction partner capping agent	Hierarchical microstructures (Kundu et al., 2012)
LaF_3_:Eu^3+^				
BaCaLu_2_F_10_:Ln^3+^ (Ln = Eu, Dy, Tb, Sm, Yb/Er, Yb/Ho)	Hydrothermal	[C_4_mim][BF_4_]	Reaction partner, solvent capping agent	Sub-microspheres (Liu et al., 2018)
CaF_2_:Ce^3+^/Mn^2+^	Hydrothermal	[C_8_mim][BF_4_]	Reaction partner capping agent	Sub-Micron cubes nanospheres (Song et al., 2012)
		[C_4_mim][PF_6_]		
YBO_3_:Eu^3+^	Hydrothermal	[C_8_mim][[Cl]	Capping agent	Microspheres (Tian et al., 2014)
FNaY(MoO_4_)_2_:Tb^3+^	Hydrothermal	[C_8_mim][[Cl]	Capping agent	Dendritic (Tian et al., 2012)
BaF_2_:Gd^3+^,Eu^3+^	Microwave	[C_4_mim][BF_4_]	Cubic	Two-Dimensional Plates Tangled (Lorbeer et al., 2014)
CaF_2_:Gd^3+^,Eu^3+^				
LaF_3_:(Dy^3+^,Tm^3+^)	Microwave	[C_4_mim][BF_4_]	Reaction partner solvent	Particles (Lorbeer and Mudring, 2013a)
NaYF_4_:Yb^3+^,Er^3+^ NaYF_4_:Yb^3+^,Tm^3+^	Microwave	[C_4_mim][BF_4_] [C_4_mim][PF_6_]	Reaction partner solvent	Nanocluster nanoparticles (Chen et al., 2010)
REF_3_ (Ln^3+^ = La to Sm)	Microwave	[C_4_mim][BF_4_]	Reaction partner solvent	Nanodisks Submicrospindles (Li et al., 2011a)
REF_3_ (Ln^3+^ = Eu to Lu, Y)				
LaF_3_:Ce^3+^,Tb^3+^	Microwave	[C_4_mim][BF_4_]	Reaction partner solvent	Uniformly ellipsoidal
LaPO_4_:Ce^3+^,Tb^3+^ and LaPO_4_:Eu^3+^	Microwave	[MeBu_3_N][(SO_2_CF_3_)_2_N]	Solvent	Spherical - ellipsoidal (Bühler and Feldmann, 2007)
LaPO_4_:Ce^3+^,Tb^3+^	Microwave	[N(*t*Bu)_3_(Me)][N(SO_2_CF_3_)_2_])	Reaction partner solvent	Spherical to Slightly ellipsoida (Bühler and Feldmann, 2006)
CaF_2_:Yb^3+^/Er^3+^	Microwave	[MeBu_3_N]	Solvent capping agent	Spherical nanocrystals & Polyhedral crystals (Zhao et al., 2015)
		[(SO_2_CF_3_)_2_N]		
BiPO4: Ln^3+^-(Ln^3+^ = Sm, Eu,Tb,Dy)	Microwave	[N_1114_][H_2_PO_4_]	Reaction partner solvent	Nanoparticles (Cybinska et al., 2016)
YPO4:Eu^3+^	Microwave	[Choline][H_2_PO_4_]	Reaction partner solvent	Nanotubes spherical particles (Cybinska et al., 2011)
LaPO_4_:Eu^3+^				
GdPO_4_: Eu^3+^				
LnPO_4_ (Ln^3+^ = Pr, Nd, Sm, Eu, Tb, and Dy)	Microwave	[N_1114_][H_2_PO_4_] [Choline][H_2_PO_4_]	Reaction partner solvent	Nanotubes (Cybinska et al., 2017)
GdF_3_:Eu@GdPO_4_	Microwave	[C_4_mim][BF_4_]	Reaction partner solvent capping agent	Brick-shaped particles (Cybińska et al., 2012)
		[Choline][H_2_PO_4_]		
LaPO_4_:Ce^3+^,Tb^3+^	Microwave	[N(*t*Bu)_3_(Me)][N(SO_2_CF_3_)_2_])	Solvent	Small particles (Zharkouskaya et al., 2008)
LaF_3_:Tb^3+^	Ultrasonic	[C_4_mim][BF_4_]	Reaction partner solvent	Nanoplates, Microcylinders (Zhu et al., 2014)
LaF_3_:Ce^3+^,Tb^3+^	Sonochemical	[C_4_mim][BF_4_]	Reaction partner solvent	Nanoparticles (Zhang et al., 2012)
CeF_3_:Tb^3+^	Ultrasonic	[C_4_mim][BF_4_]	Reaction partner solvent	Nanodisk (Liu et al., 2014b)
LaPO_4_:Eu^3+^	Microemulsion TBP/[Omim]Cl/H_2_O	([C_8_mim]-[Cl]	Solvent	Nanowires nanoparticles (Zhang et al., 2009)
CePO_4_:Tb^3+^				
α-NaYbF_4_:Gd^3+^, Tm^3+^	Two-phase system OA/ionic liquid	[C_4_mim][BF_4_]	Reaction partner solvent	Nanocrystals (Pan et al., 2013)
β-NaYbF_4_:Gd^3+^, Tm^3+^				
α-NaGdF_4_:Yb^3+^, Er^3+^	Two-phase system OA/ionic liquid	[C_4_mim][BF_4_]	Reaction partner solvent	Spherical nanocrystals (He et al., 2011b)
β-NaGdF_4_:Yb^3+^, Er^3+^				
α-NaYF_4_:Yb^3+^,Er^3+^				
β-NaYF_4_:Yb^3+^, Er^3+^				
NaGdF_4_:Yb^3+^, Er^3+^ (Ho^3+^, Tm^3+^)	Two-phase system OA/ionic liquid	[C_4_mim][BF_4_]	Reaction partner solvent	Nanocrystals (He et al., 2011a)

**(A) Hydrothermal/solvothermal**: hydrothermal or solvothermal method is rapidly used for the synthesis of Ln^3+^-doped nanoscale particles (Liu et al., 2014a; Tian et al., 2014; Wang et al., 2015b; Cybińska et al., 2016; Sharma et al., 2020a; Muthulakshmi et al., 2020; Chouryal et al., 2021). When water is used as a solvent during synthesis, this is called a hydrothermal method. However, if another solvent except water is used for synthesis, this is called a solvothermal method. In this method, the reaction mixture is transferred into the Teflon-lined vessel, further coated with a stainless steel jacket. Thereafter, the vessel is put at a particular temperature, resulting in high pressure inside the reaction vessel that accelerates the reaction. As a result, the desired product is obtained. ILs have been frequently employed in the synthesis of varieties of Ln^3+^-doped binary/ternary fluorides, phosphate, and oxides nanoparticles as ILs have high thermal and chemical stability (Liu et al., 2014a; Tian et al., 2014; Wang et al., 2015b; Cybińska et al., 2016; Sharma et al., 2020a; Muthulakshmi et al., 2020; Chouryal et al., 2021). For instance, Wang et al. have synthesized the luminescent Eu^3+^-doped LaF_3_ and YF_3_ nanoparticles using the amphiphilic diallyl dimethylammonium tetrafluoroborate ([DADMA][BF_4_]) IL using the hydrothermal method (Wang et al., 2015a). Li and coworkers have employed a similar method to prepare the water-soluble and green luminescent LaF_3_:Ce,Tb nanodisks with 25 nm size using 1-butyl-3-methylimidazolium tetrafluoroborate [C_4_mim][BF_4_] IL (Guo et al., 2010). Liu et al. have prepared the uniform LuF_3_:Ln^3+^(Ln = Eu, Tb, Dy) nanocrystals using the 1-octyl-3-methylimidazolium hexafluorophosphate ([C_8_mim][PF_6_]) *via* the solvothermal method (Liu et al., 2014a). An IL-assisted hydrothermal method has been employed to prepare BaCaLu_2_F_10_:Ln^3+^ (Ln = Eu, Dy, Tb, Sm,Yb/Er, and Yb/Ho) sub-microspheres by Liu et al. In this synthesis method, 1-butyl-3-methylimidazolium tetrafluoroborate ([C_4_mim][BF_4_])IL is used (Liu et al., 2018). Another group has synthesized the CaF_2_:Ce^3+^/Mn^2+^ sub-micro cubes and nanospheres using the 1-octyl-3-methylimidazolium hexafluorophosphate ([C_8_mim][PF_6_]) and 1-octyl-3-methylimidazolium tetrafluoroborate([C_8_mim][BF_4_]) ILs (Song et al., 2012). Yan et al. have synthesized the RE^3+^ (Eu^3+^, Yb^3+^/Er^3+^ and Yb^3+^/Tm^3+^)-doped NaYF_4_ nanocrystals using the 1-chlorohexane-3-methylimidazolium chloride ([C_6_mim][Cl]) IL-assisted hydrothermal method (Yan et al., 2014). Sharma et al. have prepared the BaF_2_:Eu^3+^ and BaF_2_:Ce^3+^ nanoparticles using the 1-ethyl-3-methylimidazolium bromide [C_2_mim][Br] and 1-butyl-3-methylimidazolium tetrafluoroborate [C_4_mim][BF_4_], respectively, via the solvothermal method (Sharma et al., 2019; Sharma et al., 2020a). Several RE^3+^-doped binary and ternary fluorides nanoparticles have been synthesized by Ghosh and coworkers using the IL-assisted solvothermal method (Ghosh et al., 2011; Ghosh and Mudring, 2016; Ghosh et al., 2017). In addition, RE^3+^-doped phosphate, oxides, oxyfluoride, and borate nanoparticles were also synthesized using the hydrothermal method. For example, Zou et al. have prepared the Ca_5_(PO_4_)_3_Cl:Ce^3+^,Tb^3+^ nanostructures with straw-like sheaves and microrod-like morphology through 1-octyl-3-methylimidazolium chloride ([C_8_mim][Cl]) IL-based hydrothermal method (Zou et al., 2013). [C_4_mim][BF_4_] IL-based solvothermal method is employed for synthesizing the upconverted Yb/Tm co-doped Y_7_O_6_F_9_ microparticles (Zhao et al., 2018). Cybinska et al. have prepared the YPO_4_:Eu^3+^ nanoscale particles using the [Choline][H_2_PO_4_] IL-assisted hydrothermal method. During synthesis, the colloidal suspension was heated at different temperatures of 100, 150, and 200°C for 10 h to obtain nanoscale YPO_4_:Eu^3+^ (Cybińska et al., 2016). Choline dihydrogenphosphate[Cholin][H_2_PO_4_] IL-assisted hydrothermal method was also employed by other groups to prepare the green-emitting Na_3_Y_1-x_(PO_4_)_2_:xTb^3+^ phosphors (Liu et al., 2020). γ-Gd_2_S_3_ nanoparticles are prepared using 1-ethyl-3-methylimidazolium ethyl sulfate ([C_2_mim][EtSO_4_]) IL-based hydrothermal method, which leads to nanoflower morphology (Khajuria et al., 2016). Tian et al. have reported the YBO_3_:Eu^3+^ and NaY(MoO_4_)_2_:Tb^3+^ phosphors using the 1-methyl-3-octylimidazolium chloride ([C_8_mim][Cl]) IL-based hydrothermal process (Tian et al., 2012; Tian et al., 2014).

**FIGURE 6:**
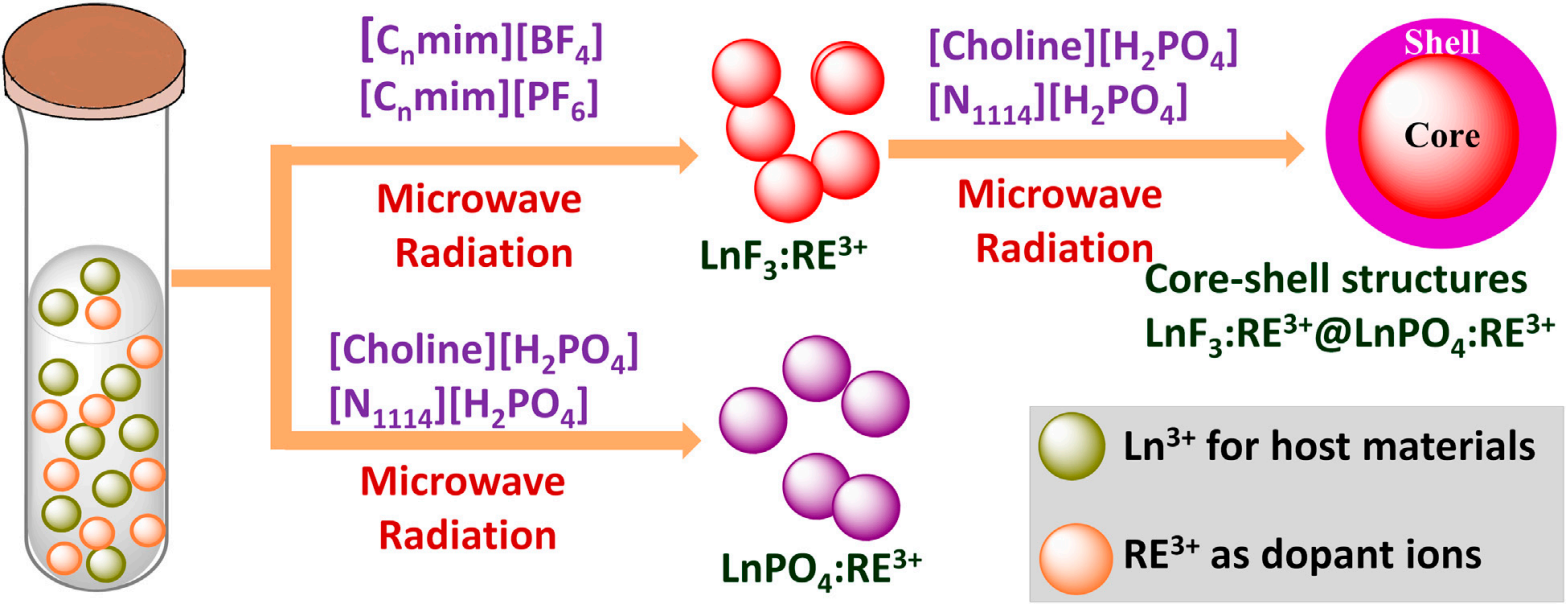
The microwave-assited IL method for synthesis of LnF_3_:RE^3+^@LnPO_4_ (Cybińska et al., 2012).

**(B) Microwave-assisted ionic liquid method**: microwave- and IL(s)-based technologies are promising green, energy-efficient, and environmentally benign methods for synthesis of nanomaterials and they have gained tremendous attention in the last decade (Wang et al., 2019). ILs have potential to absorb microwave radiation efficiently due to high polarizability (consist of large ions) and conductivity. Therefore, the combination of IL and microwave in the synthesis is also called microwave-assisted ionic liquid synthesis (MAIL) (Wang et al., 2019). Several nanostructures, including metallic, semiconductors, and metal complexes, have been prepared using microwave-assisted ionic liquid synthesis (Wang et al., 2019). This synthesis method has also been extensively employed for preparing numerous Ln^3+^-doped nanomaterials. For example, Cybinska et al. have prepared the phosphate-based nanomaterials doped with Ln^3+^ ions such as Eu-doped YPO_4_, GdPO_4_, LaPO_4_, and BiPO_4_:Eu^3+^ using the choline or butylammonium dihydrogen phosphate ([Choline][H_2_PO_4_]) IL (Cybinska et al., 2011; Cybinska et al., 2016). In addition, other LnPO_4_ (Ln = Y, La, Gd, doped with Eu^3+^ and Ln = Pr^3+^, Nd^3+^, Sm^3+^, Eu^3+^, Tb^3+^, and Dy^3+^) nanoscale particles are also prepared using the microwave-assisted method in the presence of [Choline][H_2_PO_4_] IL (Cybinska et al., 2017). During the synthesis, [choline][H_2_PO_4_] IL is utilized to absorb the microwave radiation efficiently and serves as a reaction precusor, i.e., souce of PO_4_^3−^ ions (Cybinska et al., 2011; Cybinska et al., 2016; Cybinska et al., 2017). Using the same synthesis method, fluoride nanophosphors such as GdF_3_:Eu^3+^ are synthesized using the [choline][BF_4_] IL. The as-prepared fluoride nanophosphors are further coated with GdPO_4_ using the [choline][H_2_PO_4_] IL (choline = 2-hydroxyethyl trimethylammonium), leading to the formation of oxygen-free GdF_3_:Eu^3+^@GdPO_4_ (Cybińska et al., 2012) (Figure 6). Bühler et al.have synthesized the transparent and luminescent LaPO_4_:Ce,Tb and LaPO_4_:Eu nanophosphors using the tributyl methyl ammonium triflylimide ([MeBu_3_N][(SO_2_CF_3_)_2_N]) IL as a solvent and in a laboratory microwave oven (Bühler and Feldmann, 2006; Bühler and Feldmann, 2007). Besides, the microwave-assisted IL method is also beneficial for synthesizing Ln-doped binary and ternary fluorides nanoparticles. For instance, Ln^3+^ (Ln = Eu, Gd)-doped alkaline-based binary fluorides (BaF_2_ and CaF_2_) are synthesized by Mudring and coworkers using the [C_4_mim][BF_4_] IL (Lorbeer et al., 2014). Lobreer et al. have prepared the Dy and Tm co-doped LaF_3_ nanophosphors using the [C_4_mim][BF_4_] IL (Lorbeer and Mudring, 2013a). Other nanofluorides, such as GdF_3_: Eu^3+^, NaGdF_4_:Er,Tb, Eu^3+^, and Gd^3+^ co-doped BaF_2_, triply doped LaF_3_:Ln (Ln = Tm^3+^, Tb^3+^ and Eu^3+^), and EuF_3_ nanoparticles, have been synthesized by Lobreer et al. using various types ILs via the microwave-assisted method (Lorbeer et al., 2010; Lorbeer et al., 2011a; Lorbeer et al., 2011b; Lorbeer and Mudring, 2013a; Lorbeer and Mudring, 2013b; Lorbeer and Mudring, 2014). Tessitore et al. have used ethylene glycol and various ILs for synthesizing the sub-10 nm β-NaGdF_4_:Yb^3+^,Er^3+^ nanoparticles (Tessitore et al., 2019). To determine the ascorbic acid, LaF_3_:Ce,Tb nanoparticles are prepared by Xu and coworkers in the presence of [C_4_mim][BF_4_] IL via the microwave-assisted solvothermal method (Mi et al., 2013). Furthermore, other groups have also made a significant contribution to the synthesis of luminescent binary and ternary fluorides using this method (Chen et al., 2010; Li et al., 2011a; Zhao et al., 2015).

**(C) Ionic liquid-assisted sonochemical method**: the sonochemical method for synthesizing nanomaterials is one of the pivotal methods in which ultrasound is used to induce the chemical reaction. As a result, the physical and chemical properties of the prepared nanoscale particles can be tuned (Thompson and Doraiswamy, 1999). During the sonochemical method, high-energy bubbles form, which store an enormous amount of energy in them. When those energy bubbles are collapsed, high temperature (∼5000 K) and high pressure (∼1,000 bar) are generated for a very short time, which is enough to accelerate the chemical reactions to many folds (Li et al., 2021). Along with IL, this synthesis method is often considered a green method of nanoparticles synthesis. To date, the IL-assisted sonochemical method has been used for designing the numbers of Ln-based nanophosphors materials. For example, Zhu et al. have employed this synthesis method to prepare the hexagonal LaF_3_:Tb^3+^ phosphors in the presence of [C_4_mim][BF_4_] IL and IL serve as co-solvent, capping reagent and fluoride source (Zhu et al., 2014). The small and hydrophilic LaF_3_:Ce,Tb nanoparticles with a size of less than 10 nm are obtained using the one-pot sonochemical-assisted IL method. The [C_4_mim][BF_4_] IL was employed as a fluorinating agent and ethylene glycol as a solvent (Zhang et al., 2012). By using the same [C_4_mim][BF_4_] IL, another group has synthesized the uniform size nanodisks of CeF_3_:Tb and the mean diameter and thickness of nanodisk were found to be 450 and 80 nm, respectively (Liu et al., 2014b). In addition to Ln-doped fluoride nanomaterials, the sonochemical-assisted IL method is also employed to prepare Ln-doped oxide nanomaterials. For preparing the Ln_2_O_3_:Eu^3+^ (Ln = Y, La, Gd), 1-butyl-3-methylimidazolium bistrifluoromethanesulfonyl amide ([C_4_mim][Tf_2_N]) IL was used. First, Ln(OH)_3_:Eu (Ln: Gd, La, Y) nanoparticles were found and then as-prepared nanoparticles are calcined at 800°C for 3 h to turn into Ln_2_O_3_:Eu^3+^ (Alammar et al., 2016).

**(D) Other methods*:*** in addition to previously discussed methods, additional methods have been reported to prepare the Ln-doped nanoparticles. In those methods, one or two methods have been employed to prepare the nanoparticles. IL-assisted microemulsion method is used for preparing the LaPO_4_:Eu and CePO_4_:Tb nanocrystals. The microemulsion of TBP/[C_8_mim]Cl/H_2_O was designed by properly mixing the tributylphosphate, 1-octyl-3-methylimidazolium chloride, and water. The tributylphosphate and [C_8_mim][Cl] IL were used in synthesis to control the nucleation and growth of the nanocrystals (Zhang et al., 2009). IL ([A336][cyanex272]) extraction method was employed to synthesize the LnPO_4_ (Ln = La–Gd) nanorods and also luminescence behaviors of CePO_4_:Tb nanorods were studied.

The role of IL was used to extract the LnPO_4_ into the organic phase from the aqueous phase via capping it (Zhang and Chen, 2019). Another important method in which oleic acid/ionic liquid (OA/IL, IL = [C_4_mim][BF_4_] or [C_4_mim]PF_6_]) two-phase system was utilized not only for synthesis but also for controlling the phase, size, and morphology of as-prepared Ln-doped ternary fluorides (NaYbF_4_:Gd,Tm; NaYF_4_:Yb,Er; NaYbF_4_:Er; NaGdF_4_:Yb, Er; NaGdF_4_:Yb, Ho; NaGdF_4_:Yb, Tm) (He et al., 2011a; He et al., 2011b; He et al., 2012; Pan et al., 2013). The one-step electrodeposition in IL **(**1-butyl-1-methylpyrrolidinium bis(trifluoromethanesulfonyl)imide [Py_1,4_][TFSI]) method is employed for synthesizing the luminescent silicon–terbium nanowires by Thomas et al. (2020). Another group has prepared the luminescent LaPO_4_:Ce,Tb phosphors using IL-driven liquid membrane system. The supported membrane was prepared by mixing the hydrophobic porous polyvinylidene fluoride film (HVHP) and IL ([C_4_mim][BF_4_] or [C_4_mim][Tf_2_N]) and the effect of ILs on the release of PO_4_^3-^ ion to form the LaPO_4_:Ce,Tb phosphor is also studied. IL-assisted sol-gel and leaves extract-based methods have been used for the synthesis of ZnO:Ce nanophosphors and Ln_2_O_3_ (Ln = La, Nd, Yb, Sm), respectively (Jiang et al., 2012; Muthulakshmi et al., 2020; Muthulakshmi and Sundrarajan, 2020; Veerasingam et al., 2020; Sundrarajan and Muthulakshmi, 2021). In the leaves extract-based synthesis of Ln_2_O_3_nanoparticles, *Andrographis paniculata* leaves extract is employed to prepare the Nd_2_O_3_, La_2_O_3_ and Sm_2_O_3_ nanoparticles using [C_4_mim][PF_6_]IL-assisted hydrothermal method (Muthulakshmi et al., 2020; Veerasingam et al., 2020; Sundrarajan and Muthulakshmi, 2021). In another synthesis, *Couroupita guianensis* Abul*.* leave extract is used for the synthesis of Yb_2_O_3_ nanoparticles using the [C_4_mim][BF_4_] IL-assisted hydrothermal method (Muthulakshmi and Sundrarajan, 2020).

## Intrinsic Properties of ILs That Help in Nanomaterials Synthesis

In Ln-doped nanophosphors synthesis, ILs are not only exploited as reaction media but also used as reaction partners and templating/capping agents (He et al., 2011a; Mi et al., 2013; Pan et al., 2013; Liu et al., 2014a; Cybinska et al., 2016; Liu et al., 2018; Sharma et al., 2020a; Chouryal et al., 2021). The effects of tunable properties of ILs are significantly observed on the crystal phase, host matrix, controlling the size, and modification of morphology (He et al., 2011a; Mi et al., 2013; Pan et al., 2013; Liu et al., 2014a; Cybinska et al., 2016; Ghosh and Mudring, 2016; Liu et al., 2018; Sharma et al., 2020a; Chouryal et al., 2021). In this way, by controlling all these structural properties of Ln-doped nanocrystals, the optical properties of the dopant ion can be judiciously tuned. In this section, we have emphasized the applicability of ILs in the synthesis of Ln-doped nanoparticles, synthesis of host matrix using ILs, and complex formation between IL and RE^3+^ ions, which functionally depend on the types of interactions involved between IL and RE^3+^ ions (Li et al., 2011a; Ghosh and Mudring, 2016; Prodius and Mudring, 2018).

### Reaction Medium and Capping/Templating Agent

In the beginning, ILs were used as a reaction medium for synthesizing the inorganic nanomaterials, especially lanthanide-based nanomaterials. Distinctive features of ILs, like high thermal stability, negligible vapor pressure, broad liquidus range, and most importantly adjustable properties, make them far better than conventional molecular liquids (Veselý et al., 1988; Diedenhofen et al., 2007; Ludwig and Kragl, 2007; Wang et al., 2007; Faridbod et al., 2011; Shukla and Sah, 2013). The negligible vapor pressure of ILs can be estimated by the enthalpy of vaporization, and its magnitude for ILs is much higher than the conventional liquids (Veselý et al., 1988; Ludwig and Kragl, 2007). These features of ILs increase their potential as a reaction medium.

#### (A) Tuning the Crystal Phase

Tuning of crystal phase of the nanomaterials is a state-of-the-art approach, which is substantially dependent on various factors such as nature and viscosity of reaction medium, surfactants, and varying reaction temperatures (Ghosh et al., 2008b; Duan et al., 2011; Pensado and Pádua, 2011; Song et al., 2012; Liu et al., 2014a). Amongst them, the reaction medium and its concentration have a pivotal influence on tuning the crystal phase of nanoscale particles *via* controlling the kinetics of reaction (He et al., 2012; Ju et al., 2013; Ghosh and Mudring, 2016). For instance, Lin and coworkers have synthesized the binary fluorides with the variation of crystal phase via microwave-assisted synthesis using ([C_4_mim][BF_4_])ionic liquid (Li et al., 2011a). The product and crystal phase formation was controlled based on ionic radius of RE^3+^ ion (Li et al., 2011a; Ghosh et al., 2017). Based on ionic radii of Ln-based nanoparticles, not only was the nature of products like binary (LnF_3_)/ternary fluorides (NaLnF_4_) prepared, but also the crystal phase and morphology are tuned under similar reaction conditions. In this synthesis, IL is employed as a structure-controlling agent to direct the size and shapes of the nanoparticles (Ghosh et al., 2017). He et al. have used a liquid-liquid two-phase system in which *n*-octanol induced oleic acid (OA) and IL two-phase system is used for controlling the crystal phase of RE fluorides (RE = La, Gd, and Y) (He et al., 2012). Zhong et al. have tuned the crystals of YF_3_ by just maintaining the [C_4_mim][BF_4_]/Y^3+^ ratio. The cubic phase YF_3_ particles were noticed when the molar ratio of [C_4_mim][BF_4_]/Y^3+^ was controlled to be below 0.75:1, while mixed cubic and orthorhombic phases appeared upon controlling the molar ratio to be 1:1. As the ratio is further increased to be above 1:1, pure orthorhombic phase YF_3_ particles were formed (Zhong et al., 2009). However, phase tuning of NaYF_4_ from mixed cubic to pure hexagonal phase is noticed due to an increase in the volume of IL ([C_4_mim][PF_6_]) (He et al., 2012). Alternatively, on using [C_4_mim][BF_4_] IL in place of [C_4_mim][PF_6_] IL, a significant increase in peak intensity of cubic phase of NaYF_4_ nanocrystals was noticed (He et al., 2012). Using a similar synthesis method, the same group has found that methanol has a pivotal role in phase selectivity and solubility of upconverting Ln-doped NaGdF_4_:Er-Yb nanocrystals (He et al., 2011a). In the absence of methanol, a reaction took place in oleic acid (OA), leading to the formation of OA-capped cubic phase of NaGdF_4_:Yb, Er, nanocrystals, which were dispersible in the oil phase. In contrast, IL-capped, water-soluble, hexagonal phase NaGdF_4_:Yb,Er nanocrystals was observed in [C_4_mim][BF_4_] IL phase upon adding the methanol (He et al., 2011a). However, Ju et al. have tuned the crystal phase of Ln-doped NaGdF_4_ nanocrystals via interface-assisted synthesis method by varying the polyethyleneimine (PEI) concentration and [P_66614_][PF_6_] IL. When increasing the PEI amount in the presence of IL, the crystal phase of NaGdF_4_ nanocrystals is transformed from cubic (α) to hexagonal (β) phase, whereas no formation of NaGdF_4_ occurred in the absence of PEI. However, at all concentrations of PEI, only hexagonal NaGdF_4_ nanocrystals were found using the NH_4_F in place of IL (Ju et al., 2013). The same group has again tuned the crystal phase of NaGdF_4_:Er-Yb nanocrystals using organic phase and hydrophilic [C_4_mim][BF_4_] IL system via an interface-assisted synthesis method (Ju and Mudring, 2013). On the other hand, Ghosh et al. have noticed the significant influence of pendant alkyl chain length, the interaction of crystal facet with IL *via* H bonding, and concentration of ILs on the crystal phase of the oxygen-free NaGdF_4_:Eu^3+^ nanocrystals (Ghosh and Mudring, 2016). In the synthesis, when no IL was used, only a cubic phase was found with less crystallinity; however, a hexagonal phase was noticed in the presence of [C_2_mim]Br IL under identical reaction conditions. To understand the effect of π-interaction, counter ion, and hydrogen bonding on the crystal phase, TMAB [C_2_mim][Cl] and [C_2_dmim][Br] ILs were employed, respectively. From this study, no effect of hydrogen bonding and counteranion of IL on the crsytals was found, as only hexagonal phase of NaGdF_4_:Eu(2%) nanocrystals was obtained (Ghosh and Mudring, 2016). However, in the case of TMAB, only cubic phase was observed. TMAB is employed to understand the role of non-aromatic cation of IL on the crystal phase. Besides, to get more insight into the effect of higher alkly chain length of ILs and hydrogen bonding on the crystal phase of nanocrystals, [C_4_mim][Br], [C_8_mim][Br], or [C_2_dmim][Br] ILs were employed. It is found that due to steric hindrance caused by the long alkyl chain length of ILs, unlike [EMIM][Br] IL, only cubic phase of NaGdF_4_:Eu(2%) nanocrystals was found (Ghosh and Mudring, 2016).

#### (B) Shape and Size

**FIGURE 7:**
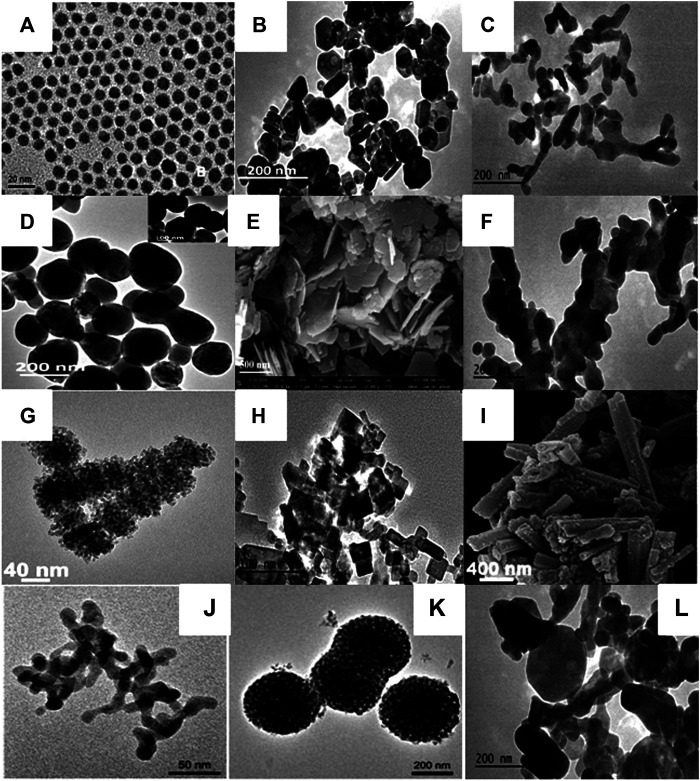
**(A)** TEM images NaYbF_4_, 2%Tm nanocrystals (Pan et al., 2013), **(B)** TEM images of the CeF_3_:Tb^3+^ (Ghosh et al., 2017), **(C)** TEM image of NaSmF_4_ (Ghosh et al., 2017), **(D)** TEM image of the NaYbF_4_:Er^3+^ nanoparticles (Ghosh et al., 2017), **(E)** SEM image of the BaF_2_:Ce^3+^(0.1%) synthesized using [C_4_mim][BF_4_] (Sharma et al., 2020a), **(F)** TEM image of NaTbF_4_:Ce^3+^ (Ghosh et al., 2017), **(G)** TEM micrographs of LaF_3_: 1% Dy^3+^,5% Tm^3+^ (Lorbeer and Mudring, 2013a), **(H)** TEM image of BaF_2_:Ce^3+^/Tb^3+^ nanoparticles [C_2_mim][Br] (Sharma et al., 2020b), **(I)** SEM image of YPO_4_ (Cybinska et al., 2011), **(J)** β-NaYF_4_:20%Yb^3+^,0.2%Tm^3+^ nanoparticles in [C4mim][PF_6_] (Chen et al., 2010), **(K)** β-NaYF_4_:20%Yb^3+^, 0.2%Tm^3+^ nanoclusters obtained in [C_4_mim][BF_4_] (Chen et al., 2010), **(L)** TEM image of NdF_3_ (Ghosh et al., 2017).

Along with crystal phase tuning, ILs are also extensively exploited as morphology-controlling and size-regulating agents due to the presence of tunable cations (alkyl chain length), hydrogen bonding, and π-stacking ability. IL's concentration also significantly affects the shape and size of nanocrystals (Kowsari and Faraghi, 2010; Ghosh and Mudring, 2016). For instance, Kowsari et al. have used the ILs as templating agents to control the morphology and size of as-prepared Y_2_O_3_ nanoparticles. Upon decreasing the concentration of IL, a flower-like array of petals with a uniform size is observed. On the other hand, irregular and crossed arrays are found at high concentrations of IL (Kowsari and Faraghi, 2010). Lin and coworkers have synthesized various binary fluorides LnF_3_ nano-/microcrystals with different morphology using the [C_4_mim][BF_4_] IL. In this synthesis, the morphology of binary fluorides is changed from nanodisks (thickness = 22 nm and dia. = 55 nm) to elongated nanoparticles (length = 710 nm and dia. = 350 nm) of various sizes. The effects of different IL-based fluorinating agents on the morphology of Ln-based binary fluorides, such as CeF_3_ nanodisks, which were prepared using the [C_8_mim][BF_4_] IL, were studied; the morphology of CeF_3_:Tb^3+^ nanocrystals was found to be donut-shaped in the presence [C_8_mim][PF_6_] (Zhang et al., 2008). The effects of the counterpart of ionic liquid, i.e., BF_4_^−^ and PF_6_^−^, on the morphology of Yb^3+^/Tm^3+^ co-doped NaYF_4_ are also observed. As the [C_4_mim][BF_4_] and [C_4_mim][PF_6_] ILs were employed, not only was Yb/Tb co-doped NaYF_4_ obtained, but also morphology was tuned from nanoclusters to nanoparticles (spherical to ellipsoidal), respectively (Chen et al., 2010). Similarly, the effects of anion moieties of BF_4_^−^ and PF_6_^−^ ILs on the morphology of binary fluorides are reported (Zhong et al., 2009; Song et al., 2012). In addition, a noticeable effect of IL and TBP (tributylphosphate) on the morphology of RE ion-doped REPO_4_ (RE = La-Tb) was found. Nanocrystals and nanowires were formed in the TBP-capped and uncapped rare-earth phosphate, respectively (Zhang et al., 2009). The templating effect of [C_8_mim][Cl] IL was noticed on the morphology of YBO_3_:Eu^3+^ nano-/microstructures. Under the same pH conditions, morphology is found to be flower-like in the absence of IL, while as the IL was used, morphology turned out into a tire-like structure (Tian et al., 2014). In another synthesis, the same group has synthesized the dendrite-like NaY(MoO_4_)_2_:Tb^3+^ phosphor in the presence of [C_8_mim][Cl] IL (Tian et al., 2012). Sanxi et al. have synthesized the CeF_3_:Tb^3+^ nanodisk with the thickness of 60–65 nm and diameter of 260–425 nm using the [C_4_mim][BF_4_] IL. The formation of nanoparticles or nanodisk of CeF_3_:Tb^3+^ was dependent on the method of synthesis (Liu et al., 2014b). Kundu et al. have studied the influence of IL ([C_4_mim][BF_4_]) concentration on the morphology of LaF_3_: Ln^3+^. It was found that upon increasing the concentration of IL from 0.5 to 1.25 mmol under similar reaction conditions, the uniformity of the spheres increases (Kundu et al., 2012). Furthermore, several morphologies, such as sheaves like Ca_5_(PO_4_)_3_Cl:Ce^3+^,Tb^3+^ (Zou et al., 2013), square-shaped Y_2_O_3_,^87^ spindle-shaped Na_3_Y0_.78_(PO_4_)_2_:0.22Tb^3+^, (Liu et al., 2020), BaCaLu_2_F_10_:Ln^3+^ (Ln = Eu, Dy, Tb, Sm,Yb/Er, Yb/Ho) submicrospheres,^35^LnPO_4_ nanorods (Zhang and Chen, 2019), are reported for Ln-doped phosphors materials (Figure 7 and Table 2).

## Mechanism Related to Different Morphologies Synthesized *via* Ionic Liquids

The morphology of the as-prepared nanoparticles fundamentally depends on the nucleation and subsequent growth of the particles. For controlling the nucleation and growth, surface active agents, such as ILs, EDTA, TBP, and long-chain amines like olylamines, can play a crucial role. ILs can tune the morphology of the nanomaterials and their possible mechanisms are given as follows.

**(i) Relative reactivity of ILs**: it is already discussed that ILs can also be used as precursors to synthesize fluorides-based nanoparticles. However, releasing of F- ions by ILs in the reaction is slow and it depends on the type of the counteranions. It has been reported that thermal degradation of ILs consists of PF_6_^−^ ions as counterion is found easier than the BF_4_^−^ ions because the bond strength of P-F is weaker than the B-F bond strength (Chen et al., 2010). This characteristic of ILs enables the control of the morphology of nanoparticles. The slow release of fluoride ions by these counterions significantly influences the morphology (Chen et al., 2010; Kundu et al., 2012). Moreover, another important physical property is the viscosity of ILs, which can also play a vital role in controlling the morphology of nanoparticles. Normally, with greater viscosity of IL, less aggregated nanoparticles are formed (Chen et al., 2010).

**(ii) Hydrogen bonding and π-π stacking**: the cation moiety of ILs like [BMIM]+ consist of a hydrogen atom at the C2 position of imidazolium ring, which may form hydrogen bonding with the primary nuclei of oxide or other moieties, which eventually decides the morphology or the crystal phase of the nanocrystal. As a result, ILs can bind at the highly active site of growing nanoparticles, leading to controlled growth (Sun and Zheng, 2010). ILs are different cation/anion combinations, which can act as a soft template that attaches to the growing inorganic surfaces. Imidazolium rings of some ILs are aggregated *via* π-π stacking in the aqueous medium in such way that the formation of IL/water microemulsion occurs like other surfactants (Zhang et al., 2009; Sun and Zheng, 2010; Li et al., 2011a). As a result, spherical-shaped nanoparticles may occur inside the “nanoreactor” caused by this microemulsion.

**(iii) Adsorption at nucleation site:** Another way of controlling the morphology of nanoparticles by various surface active agents like ILs is the adsorption at the highly energetic facet of nuclei (Zhong et al., 2009; Zhang et al., 2009; Guo et al., 2010; Sun and Zheng, 2010). Consequently, the growth of nanoparticles from that particular facet decides the fate of various morphologies like the formation of 2D morphology (flat disk) and nanorods.

### Reaction Partner

Another important feature of ILs is their use as reaction partners or precursors. This characteristic of ILs makes them much superior to other traditional molecular solvents. For this, all credit is given to the tunable properties of ILs, especially anion counterions. By varying the anion part of ILs, different reaction partners can be designed according to the desired product (He et al., 2011a; Li et al., 2011a; He et al., 2011b; Song et al., 2012; Cybinska et al., 2016; Cybińska et al., 2016; Liu et al., 2018).

#### (A) Host Material Synthesis

Synthesis of nanoscale host materials using ILs is mostly dependent upon the strategy used. As aforementioned in the earlier section, fluoride-based host materials are considered as better host materials for doping of RE^3+^ ions than the other materials such as oxides and phosphates. Therefore, for the preparation of fluoride-based especially binary and ternary rare-earth-doped fluorides and phosphate-based host materials, BF_4_^−^, PF_6_^−^ ions, and H_2_PO_4_^−^ containing ILs have been extensively studied by several research groups (Zhong et al., 2009; Guo et al., 2010; Cybinska et al., 2011). These counterions of IL on heating at a particular temperature release the fluoride ions and phosphate ions in the reaction medium in order to form fluoride and phosphate-based nanomaterials. The mechanism of releasing F^−^ ion and phosphate ion during the synthesis of nanomaterials is shown as follows (Zhong et al., 2009; Guo et al., 2010; Cybinska et al., 2011):

$${\text{BF}}_{4}^{-}+3H_{2}O\to H_{3}BO_{4}+3HF+F^{-}$$

$$PF_{6}^{-}+4H_{2}O\to H_{3}PO_{4}+5HF+F^{-}$$

$$M^{2+}/Ln^{3+}+F^{-}\to MF_{2}/LnF_{3}/MLnF_{x}$$

$$Ln^{3+}+\left[Choline\right]\left[H_{2}PO_{4}\right]\to LnPO_{4}↓+2H^{+}↑+{\left[Choline\right]}^{+}$$

Frequently, imidazolium-based ILs possessed these counterions for hydrolysis. Another host material for the doping of the RE^3+^ ions is the phosphate-based LnPO_4_ nanomaterials (Cybińska et al., 2012; Cybinska et al., 2016; Cybińska et al., 2016; Cybinska et al., 2017). LnPO_4_ are also considered important host materials due to their high thermal, mechanical, physical, chemical stability and extreme resistance to oxidation and high-energy radiation sources. Therefore, LnPO_4_ nanomaterials are also substantially synthesized for doping of RE^3+^ ions. Earlier, orthophosphoric acid, tributyl phosphate, ammonium phosphate, and pyridinium phosphate were used along with ILs (templating or reaction medium) for synthesizing the LnPO_4_. However, Mudring and coworkers have first synthesized the phosphate-containing ILs and then utilized them for the synthesis of several LnPO_4_ such as choline dihydrogen phosphate ([Choline][H_2_PO_4_]) and butylammonium dihydrogen phosphate (Cybinska et al., 2016; Cybińska et al., 2016). For example, Cybinska et al. have synthesized phosphate-based nanoparticles such as Eu-doped YPO_4_, LaPO_4_, and GdPO_4_ using the [Cholin][H_2_PO_4_] IL (Cybińska et al., 2012; Cybinska et al., 2016; Cybińska et al., 2016; Cybinska et al., 2017). Other host materials were also synthesized using the ILs as reaction partners. Apatite materials [M_5_(PO_4_)_3_X, M = alkaline earth metals and X may be halogens or OH] are also considered host materials for doping of RE^3+^ ions. Thus, Zou et al. have also used [C_8_mim][Cl] IL as a source for Cl^−^ ion for the synthesis of Ce^3+^ and Tb^3+^-doped Ca_5_(PO_4_)_3_Cl nanostructures (Zou et al., 2013).

#### (B) Complex Formation With Ln ions

Rare-earth (RE^3+^) ions in +3 oxidation state also have the potential to form complexes with organic ligands or chelating agents. Typically, coordination number (C.N.) 8 or 9 is noticed for the RE^3+^ ions due to their large ionic radius. As a result, a large plethora of rare-earth complexes have been reported so far (Mudring et al., 2005; Bünzli, 2010; Prodius and Mudring, 2018). In 1942, Weissman studied the fluorescence behavior of RE complexes with organic compounds in which the excitation energy is absorbed by the organic moieties, and their excitation efficiencies were dependent on various factors such as temperature, nature of the organic compound and solvent (Weissman, 1942). The emission intensity was enhanced by many folds compared to that reported for solely RE ions, which was attributed to an intramolecular energy transfer from organic compounds to the centered RE^3+^ ions. Thereafter, several scientific groups have studied the energy process from ligand to RE^3+^ ions (Binnemans, 2009). The RE^3+^ ions cover a wide range of excitation and emission radiation, i.e.,. from ultraviolet to near-infrared region, which is typically the function of RE^3+^ ions themselves. Due to the lower absorption coefficient of RE^3+^ ions, these organic moieties act as sensitizers to absorb the energy and then transfer it to the emission center via antenna effect (Weissman, 1942; Binnemans, 2009). In the last few decades, enormous lanthanide organic complexes-based hybrid materials have been synthesized for widespread applications (Bünzli, 2010; Prodius and Mudring, 2018). In this section, we will focus only on RE-IL-based complexes and RE-containing ionic liquid synthesis. In the early stage of application, ILs were used to separate rare-earth ions from their minerals. Anions with high polarity and weak coordinating nature are more susceptible to form a complex with RE ions. Another key feature is that if the rare-earth complex has a similar anion as in IL, RE can be easily incorporated into the matrix of ILs (Prodius and Mudring, 2018). To date, numbers of anions have been identified which are frequently used to form the rare-earth complexes and rare-earth containing ILs, including halides, perfluorinated based complexes, β-diketonates, thiocyanates, nitrates, carboxylates, and polynitriles. These anions can easily form complexes with rare-earth ions and serve as counterions of ILs (Prodius and Mudring, 2018). For instance, perfluorinated anions and their derivative anions such as bis(trifluoromethylsulfonyl)imide [(CF_3_SO_2_)_2_N]^−^, which are commonly abbreviated as [Tf_2_N]^-^ [TFSI]^-^ or triflate [CF_3_SO_3_]^−^, are indispensable and extensively used as anions to construct the room temperature ILs and form the complexes with RE ions (Prodius and Mudring, 2018). Specifically, this anion does not form hydrogen bonding with the cationic part of ILs in crystals; therefore, these are either weakly coordinating or even non-coordinating in nature (Prodius and Mudring, 2018). Mudring and coworkers have made huge contributions in developing this field (Prodius and Mudring, 2018). This group has successfully prepared numerous rare-earth-based ILs using these anions. It is well known that RE^3+^ ions have different coordination abilities with ligand, including triflimidate-based ILs, which is the function of the size of RE^3+^ ions; thus, based on their size, they are divided into two classes: the large-sized RE^3+^ ions (RE = Pr–Tb) tend to form [C_4_C_1_pyr]_2_[RE(Tf_2_N)_5_], whereas the small-sized RE^3+^ ions (RE = Dy–Lu) form [C_4_C_1_pyr][RE(Tf_2_N)_4_] (Babai and Mudring, 2005; Babai and Mudring, 2006). Another widely studied ligand to form the stable complex with the RE^3+^ ions is β-diketonate and its derivatives, which have widespread applications. However, the major issue with this ligand is its high sensitivity to water, which even results in its partial or complete decomposition. This problem was overcome using the derivatives of 1,3-diketonate like fluorinated β-diketonate, 2-thenoyltrifluoroacetonate (TTA), 2-naphtoyltrifluoroacetonate (NTA), and hexafluoroacetylacetonate (HFA) (Goossens et al., 2009; Lunstroot et al., 2009; Prodius and Mudring, 2018). When utilizing the 2-thenoyltrifluoroacetonate (TTA) ligand to form the complex with Eu^3+^ ions, ethanol-/water-stable [Eu(TTA)_4_]^-^ is formed. In contrast, Tang and Mudring have used the HFA ligand and obtained the two new complexes of Tb^3+^ ions [C_1_C_4_Py][Tb(HFA)_4_] and [C_1_C_4_im] [Tb(HFA)_4_] in different ILs under similar reaction conditions ([C_1_C_4_Py]Br and [C_1_C_4_im]Cl), respectively (Mudring and Tang, 2010). In this way, several rare-earth-containing ILs were also synthesized for various applications (Prodius and Mudring, 2018).

### Influence of ILs on the Optical Properties of Ln^3+^-Doped Nanoparticles

The optical properties of Ln^3+^-doped nanoparticles are considerably influenced by ILs and other surface active agents. There are various factors such as concentration of IL, physicochemical properties of ILs like viscosity, the ratio of IL/RE^3+^ ions, which affect the crystal phase and morphology and subsequently optical properties of particles. For instance, Chen et al. have studied the effect of morphologies obtained by employing the different ILs ([C_4_mim][BF_4_] and [C_4_mim][PF_6_]) on the upconversion emission of α-NaYF_4_:20%Yb^3+^, 2%Er^3+^, and α-NaYF_4_:20%Yb^3+^, 0.2%Tm^3+^ nanoparticles and nanoclusters (Chen et al., 2010). They noticed that upconversion emission intensity of nanoclusters obtained by utilizing the [C_4_mim][BF_4_] IL was found to be nearly 8 times more intense than the emission intensity of nanoparticles obtained in the case of [C_4_mim][PF_6_]. This is due to the significant decrease of surface defects for nanoclusters compared to the nanoparticles (Chen et al., 2010). Zhao et al. have illustrated the effect of IL on the luminescence behavior of Y_2_O_3_:Yb^3+^/Tm^3+^. In this study, the upconversion emission efficiency of Y_7_O_6_F_9_:Yb^3+^/Tm^3+^ microspheres synthesized using the high concentration of [C_4_mim][BF_4_] IL is much stronger than that of Y_2_O_3_:Yb^3+^/Tm^3+^ microspheres synthesized without IL (Zhao et al., 2018). The molar ratio of [C_4_mim][BF_4_]/Ln^3+^ also brings about a good effect on the emission of YF_3_ doped with Eu (5%) (Zhong et al., 2009). The study suggests that by increasing the molar ratio up to 10.75, the emission intensity of (^5^D_0_-^7^F_1_) transition centered at 594 nm of Eu^3+^ ions is enhanced due to an increase in the crystallinity of nanoparticles. However, the formation of mixed-phase Eu-doped YF_3_ was obtained when the molar ratio of [C_4_mim][BF_4_]/Ln^3+^ increased to 1:1. By further increasing the molar ratio up to 2:1 and 4:1, emission intensity is again enhanced, which could be attributed to the formation of single-phase Eu-doped YF_3_ (Zhong et al., 2009). In another study, morphology-dependent luminescence behavior of Ca_5_(PO_4_)_3_Cl:Ce^3+^,Tb^3+^ nanostructures has been illustrated (Zou et al., 2013). It has been reported that upon increasing the [C_8_mim]Cl IL concentration, sheave- and microrod-like morphologies are observed. To understand the effect of different morphologies on the luminescence pattern, the authors have found that luminescence intensity of Ca_5_(PO_4_)_3_Cl:Ce^3+^,Tb^3+^ microrods is stronger than that of Ca_5_(PO_4_)_3_Cl:Ce^3+^,Tb^3+^ sheaves on exciting at 299 nm (Zou et al., 2013).

## Application of RE-Doped Nanomaterials

This section is typically focused on the wide range of applications of rare-earth-doped nanophosphors materials. Judicious doping of rare-earth ions in the host matrix develops new properties or property combinations for specific applications. Herein, utilization of Ln-based nanophosphors in photonic and biophotonic applications is elucidated (Gai et al., 2014; Sharma et al., 2017a).

### White Light Emission

Recently, environmentally benign white light-emitting nanophosphors have drawn considerable attention for reducing power energy consumption. Today, we have almost replaced normal incandescent lamps with compact fluorescent lamps (CFLs) or light-emitting diodes (LEDs) as both the CFLs and LEDs consume less energy than the incandescent lamps. However, like others, CFLs also use Hg (mercury) as a discharge medium, which has both environmental and health issues. Two approaches are currently being used to obtain white light: one using the combination of blue, green, and red LEDs, and another is phosphor-converted LEDs (pc-LEDs), which very much resemble the CFLs (Sharma et al., 2017a). However, LEDs and CFLs have a number of problems due to their complex construction protocols. In addition, the extreme level of purity required for phosphor materials and dimming reduces their applications to a large extent (Sharma et al., 2017a). Another important problem concerned with the generation of white light is using the combination of three different colors of light, such as blue, green, and red sources, but each component has a different lifetime. Sometimes, emitted blue light is reabsorbed by the green- and red-emitting phosphors (Gao et al., 2011). In this regard, RE^3+^-doped nanophosphors have shown interesting features, which make them more suitable candidates for this application. In addition, white light-emitting materials can also be synthesized by judiciously doping different RE^3+^ ions in the single host matrix. For instance, Mudring and coworkers have developed the white light-emitting nanophosphors simply by doping with 1% Eu, 1% Tb, and 1% Tm in LaF_3_ nanoparticles (Lorbeer and Mudring, 2013b). Upon exciting at λ_ex_ = 355 nm, white emission was obtained, which was close to the standard D65 daylight. It is believed that doping of more than one optically active dopant ion in a host matrix results in concentration-dependent quenching of emission light (Gao et al., 2011). This problem was solved by simply doping two RE^3+^ ions such as Dy^3+^ and Tm^3+^ in host materials, and in this case, white light was produced by combining their emitted complementary colors (Lorbeer and Mudring, 2013a).

### Enhancing the Photovoltaic/Solar Cell Efficiency

**FIGURE 8:**
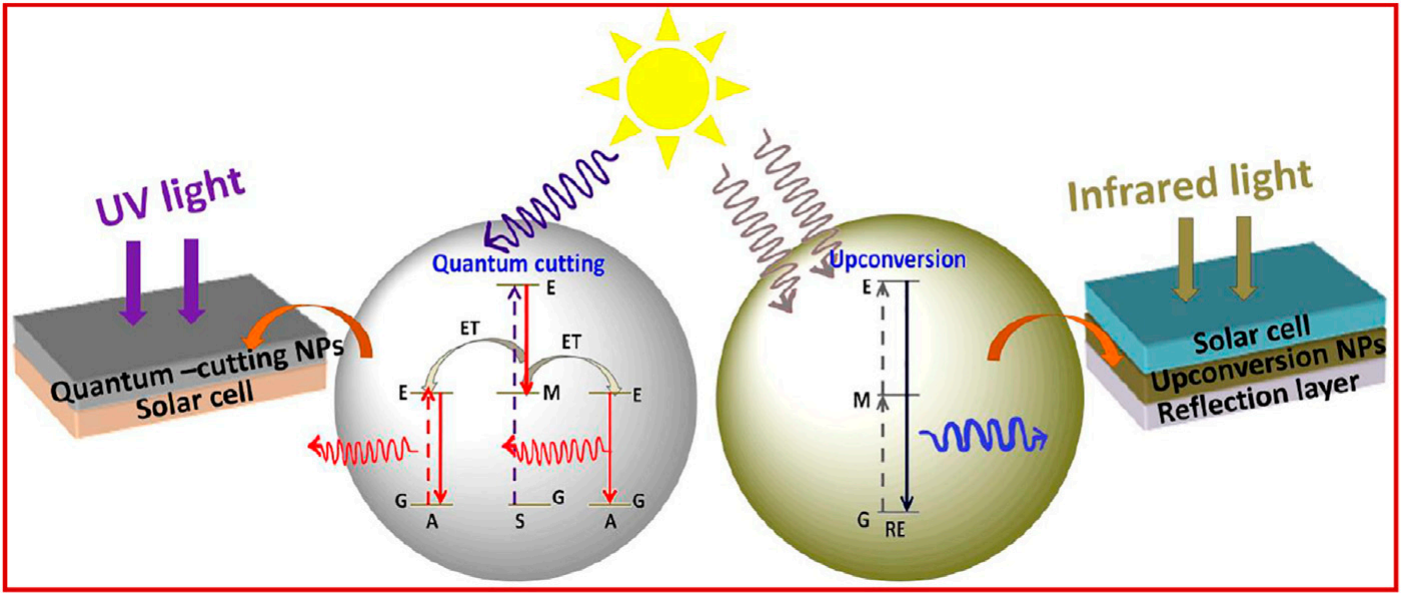
Schematic representation of quantum cutting and upconversion nanoparticles in a solar cell.

A solar cell is an important device to convert incident solar energy to useful electrical or other forms of energy. However, the major demerit of this device is the limiting bandgap. If the incident energy would be less than the bandgap of the solar device, that energy is lost, which is also known as sub-bandgap losses (Goldschmidt and Fischer, 2015; Sharma et al., 2017a). This sub-bandgap loss varies for different materials utilized in solar cell applications. For instance, more than 19% sub-bandgap loss is obtained for silicon-based solar cells (Goldschmidt and Fischer, 2015; Sharma et al., 2017a). On the other hand, in the case of gallium arsenide thin or perovskite-based solar cells, this sub-bandgap loss is reported to be from 30 to 50%, which is attributed to the associated bandgaps (Goldschmidt and Fischer, 2015; Sharma et al., 2017a). Sub-bandgap loss of solar cells can be overcome by using upconversion materials. Upconverting materials can be applied with the aforementioned solar devices to absorb the sub-bandgap lost energy. It is well known that upconverting nanomaterials are more susceptible to absorb and get excited by absorbing the NIR region of light. It has already been established that the crystal field of host material about the lanthanide ion is further split into ^2S+1^L_J_ levels into the crystal field component (Goldschmidt and Fischer, 2015; Sharma et al., 2017a). Due to this splitting, a broadening in the energy level of ^2S+1^L_J_ occurred, which means it has a broad absorption spectrum, which is an important prerequisite for photovoltaic applications (Goldschmidt and Fischer, 2015). However, the upconversion process using the non-coherent sources like the Sun is becoming more complicated than when using a coherent source due to their multistep process. Trupke and Green have proposed the theoretical relation of upconversion with the photovoltaic devices and stated that the maximum power conversion efficiency can be achieved up to 47.6% using the ideal upconverting nanomaterials on the rear side of the solar cell with the bandgap of 2eV under the non-coherent sunlight (Figure 8) (Trupke et al., 2002a). The most frequently studied RE-doped upconverting nanomaterials for solar cell applications are ternary rare-earth fluorides (NaREF_4_, especially in β-phase), rare-earth oxides (RE_2_O_3_), and oxysulphides (RE_2_O_2_S) and glass and ceramic materials. Shalav et al. (2005) have employed the NaYF_4_:Er^3+^ (20%) upconverting phosphors for enhancing the internal quantum efficiency of silicon solar cells up to 3.8% (Shalav et al., 2005). In addition, Trupke et al. have shown that downconverting materials can also be utilized to enhance the solar cell efficiency by absorbing the high-energy photons, which have energy twice the bandgap of solar cells (Figure 8) (Trupke et al., 2002b). To improve the solar cell efficiency with a bandgap of about 1.1 eV, luminescence downconverter materials (i.e., RE^3+^-doped and quantum well heterostructures) with intermediate or interband transition can play a crucial role too (Trupke et al., 2002b). For example, the upper limit of 39.63% for *Eg =*1.05 eV was calculated in which the luminescence converter had one intermediate level. Additionally, when applying the downconverter on the front surface, solar cell efficiency 38.6% for *Eg* = 1.1 eV was achieved over the conventional solar cell for which 30.9% efficiency was reported (Trupke et al., 2002b).

Furthermore, other rare-earth containing materials are also being used for increasing the solar cell efficiency like metal-organic framework with RE^3+^ ions, dye-sensitized solar cells with rare-earth materials, and perovskite materials doped with luminescent RE ions (Goldschmidt and Fischer, 2015; de la Mora et al., 2017; Zhou et al., 2017; Yu et al., 2018).

**Environmentally benign lighting and quantum cutting nanomaterials**: almost 19% of the total energy is consumed for lighting worldwide.

To fulfill the demand of energy requirement, especially in both developed and developing countries, is a great challenge due to limited conventional energy resources. Currently, Hg-based compact fluorescent lamps are used in place of traditional incandescent lamps, which have numerous issues such as slow start-up time, environmental issues, and hazardous effects on human health and disposal problems at the end. Nowadays, it can be envisaged that noble elements like xenon, which is non-toxic, can be used in CFLs as discharge media in lieu of Hg, though Xe also has its own limitations like less discharge efficiency than mercury. In order to surmount these problems, rare-earth-doped quantum cutting phosphor nanomaterials can be employed, which can convert the UV or VUV region of light into visible light (Lorbeer et al., 2011a; Ghosh et al., 2011; Ghosh and Mudring, 2016; Chouryal et al., 2021). Mudring and coworkers have shown that using the quantum cutting nanomaterials, quantum efficiency can be achieved approximately up to 200%, which is very close to the maximum possible theoretical limit (Lorbeer et al., 2011a; Ghosh et al., 2011). Ghosh et al. have reported the crystal phase-dependent quantum cutting efficiency for NaGdF_4_:Eu^3+^ nanoparticles is 154 and 107% for hexagonal and cubic phases, respectively (Ghosh and Mudring, 2016).

**FIGURE 9:**
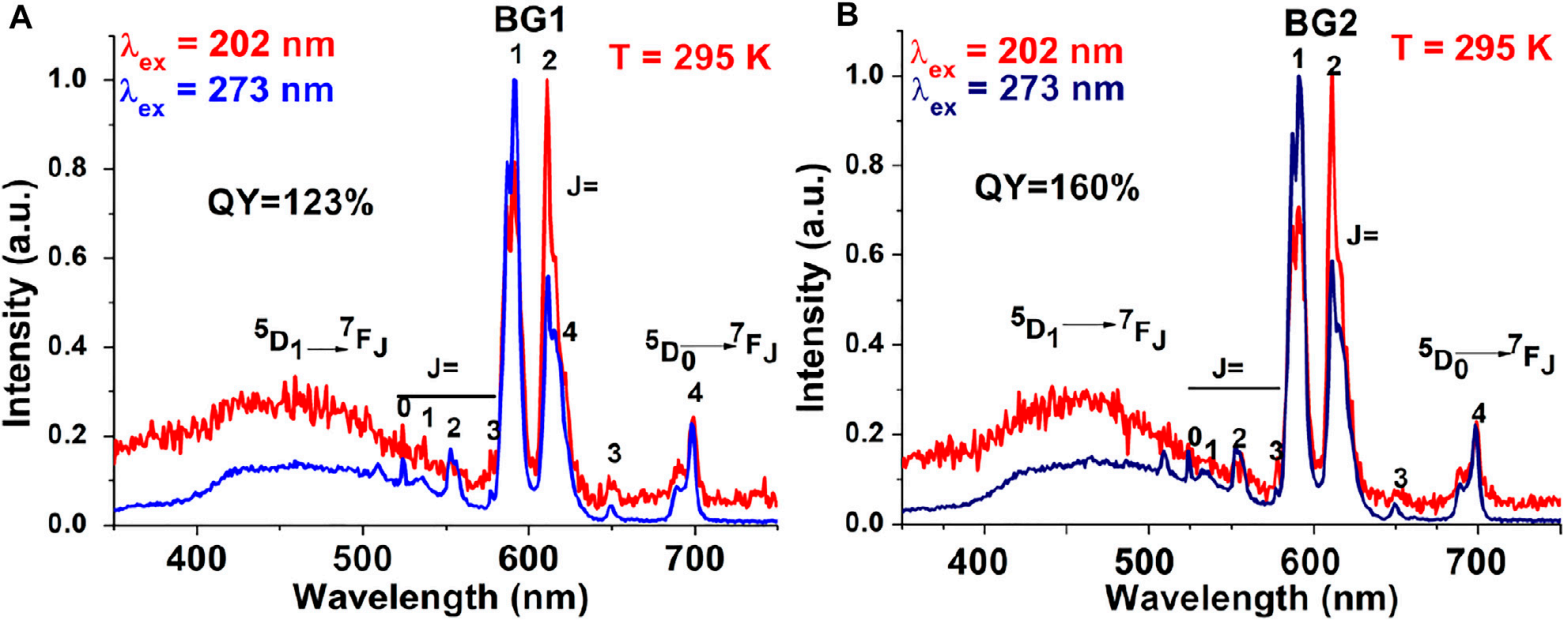
Emission spectra recorded at RT for the as-prepared BaGdF_5_:Eu^3+^ NPs synthesized in the absence **(A)** and presence **(B)** of IL (Chouryal et al., 2021).

Moreover, Chouryal et al. have also studied temperature-dependent quantum cutting behavior of as-prepared BaGdF_5_:Eu^3+^ nanoparticles (Figure 9). At room temperature, 123 and 160% quantum cutting efficacies are observed for nanoparticles that are synthesized without (BG1) and with (BG2) IL, respectively. However, at a low temperature of about 10 K, no quantum cutting is observed due to the presence of inherent Eu^2+^ ions along with Eu^3+^ ions (Chouryal et al., 2021).

### Bioimaging

Bioimaging is a powerful tool for biomedical research and clinical diagnostics applications. The prerequisite condition for bioimaging is the judicious selection of luminescent nanomaterials (Gai et al., 2014). It has been reported that several issues are associated with the application of nanoparticles that are excited by high-energy UV light due to the photodamage of living organisms and less tissue penetration depths. Due to these reasons, Ln^3+^-based downconverting nanomaterials are seldomly used for biomedical applications (Gai et al., 2014).

On the other hand, upconverting nanomaterials, especially those emitted in the range of 700–1,000 nm, have drawn tremendous attention for biomedical and clinical diagnostics applications due to low-energy excitation wavelength and high tissue penetration depth (about 1 cm) (Gai et al., 2014). Using the upconverting nanoparticles, various imaging techniques such as upconversion imaging, tumor targeting and imaging, and multimodal imaging have been developed (Gai et al., 2014). For example, water-soluble NaGdF_4_:Yb,Er upconverting nanoparticles have been used for *in vivo* dual-modality imaging in which these nanoparticles were employed for upconversion imaging and as a contrast agent (He et al., 2011a). Previously, it was found that Ln (Ln^3+^ = Yb, Gd) element having a large atomic number can be utilized as a contrast agent because these elements exhibit a high X-ray absorption coefficient (e.g., Yb, 3.88 cm^2^ g^−1^; Gd, 3.11 cm^2^ g^−1^ at 100 keV). (Gai et al., 2014).

**FIGURE 10:**
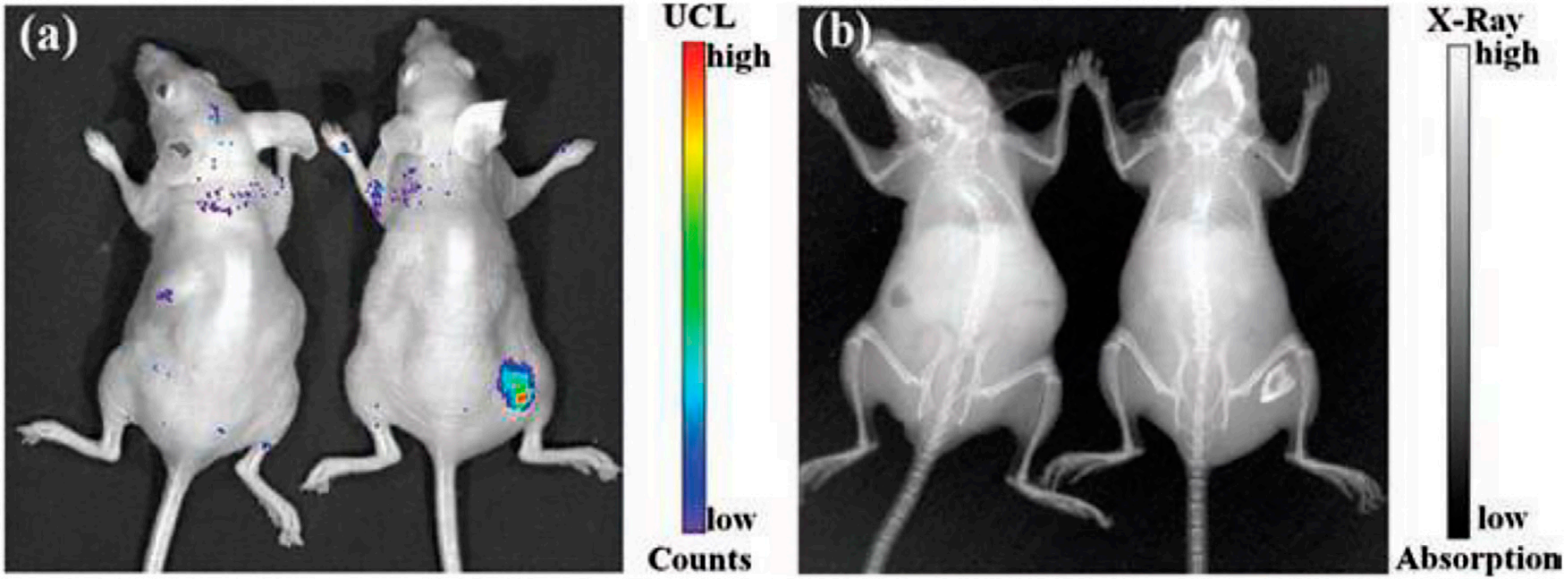
*In vivo* upconversion luminescence images **(A)** and X-ray imaging **(B)** of mice after subcutaneous injection (left) without and (right) with NaGdF_4_: Yb, Er (He et al., 2011a).

**FIGURE 11:**
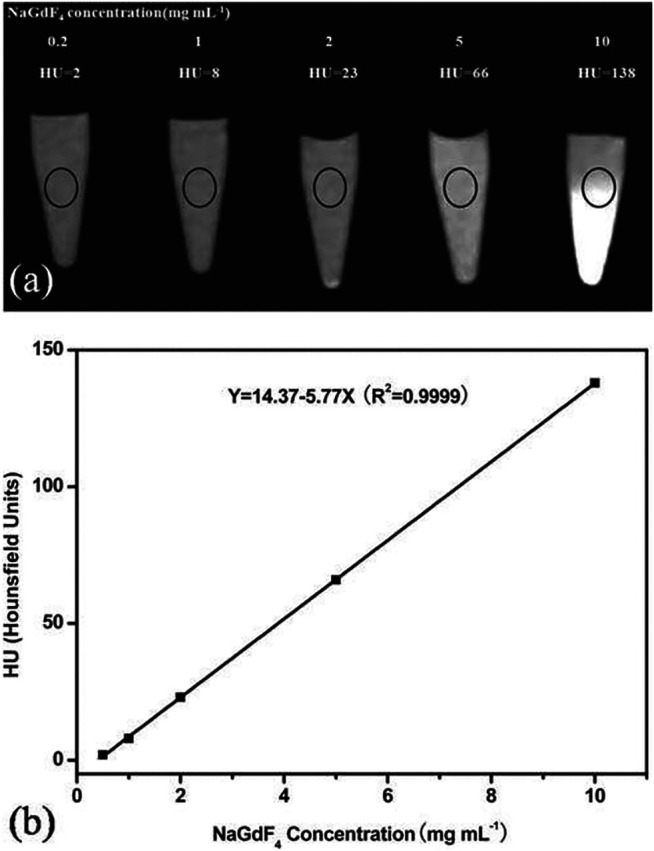
**(A)***In vitro* CT images of lanthanide-doped NaGdF_4_ upconversion nanocrystals suspended in PBS. The concentration (mg ml^−1^) in each sample is provided at the top of the respective images. **(B)** CT attenuation (HU) of lanthanide-doped NaGdF_4_ upconversion nanocrystals at various concentrations (He et al., 2011a).

Therefore, NaGdF_4_:20%Yb, 2%Er nanoparticles were injected into the body of nude mice with a concentration of 100 µL 1 mg ml^−1^ per animal. Upon irradiating the mice using the infrared laser at 980 nm, a prominent upconversion signal from the subcutaneous site was observed, whereas such signal was not found in the control mice (Figure 10A) (He et al., 2011a). Furthermore, X-ray attenuation was measured in the nude mice, and in the presence of Gd element, higher attenuation coefficient was observed due to high atomic number and electron density (Figure 10B) (Popovtzer et al., 2008). The prerequisite condition that makes them an effective contrast agent in the X-ray-based computed tomography (CT) has to be prolonged presence in the blood vessels (Janib et al., 2010). Therefore, upconverting nanoparticles have exhibited superior potential for CT imaging techniques to conventional contrast agents. Figure 11 depicts that the CT image of upconversion nanoparticles increased with increasing the mass concentration of nanoparticles. Similarly, attenuation value (HU) is also gradually increased with the concentration of Ln-doped NaGdF_4_ upconverting nanoparticles from 0.5 to 10 mg ml^−1^.

### Interaction of RE^3+^-Doped Nanoparticles With Zebrafish Biomarkers

**FIGURE 12:**
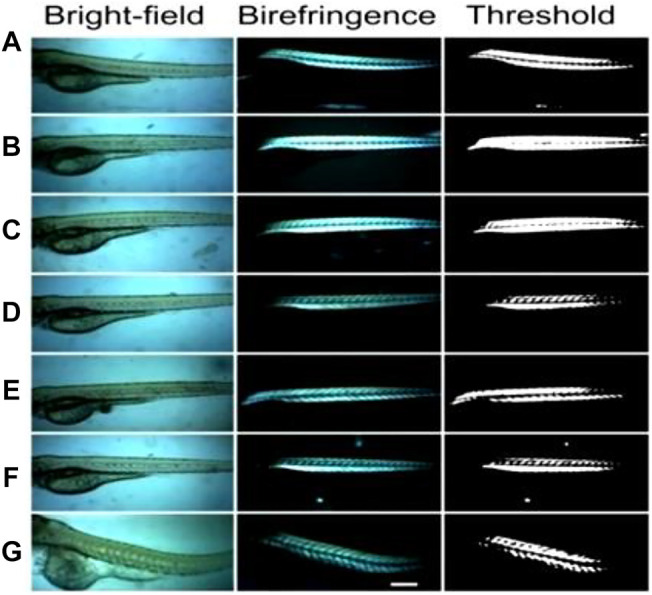
Assessment of developmental toxicity in zebrafish larvae. Optical images of zebrafish larvae at 96 hpf for Control **(A)**, DMSO **(B)**, BaF_2_ **(C)**, BaF_2_:Ce^3+^/Tb^3+^ **(D)**, BaF_2_:Ce^3+^/Tb^3+^@SiO_2_ **(E)**, BaF_2_:Eu^3+^ **(F)**, and cypermethrin pesticide **(G)**. Bright-field images showing complete skeletal muscles, yolk sac, yolk tube, and pericardial area of zebrafish larvae. Birefringence images showing the structural integrity of skeletal muscles of zebrafish larvae for the respective treatment group. Threshold images are binary birefringence images showing the area of zebrafish larvae having bright pixels falling within a pixel intensity gate of 100–255 values. Scale bar = 0.2 mm (Sharma et al., 2020b).

Recently, nanoparticles are substantially used in nanotechnology and sometimes they are directly discarded into water bodies, leading to the occurrence of water pollution. This pollution affects the aquatic systems and has hazardous effects on human beings. In addition, today, we are frequently using rare-earth-doped nanoparticles for different kinds of bioimaging, drug delivery, and other purposes. Thereby, it is very important to know the safer doses of rare-earth-doped nanoparticles used for human beings. In order to understand the toxicity effect of as-prepared RE^3+^-doped nanoparticles on the aquatic living system, zebrafish as a model is chosen (Sharma et al., 2020b). Sharma et al. have studied the effect of as-prepared RE^3+^-doped nanoparticles on the developing zebrafish larva using bright-field and birefringence imaging techniques. It is noticed that no deformation in skeletal muscles, yolk sac, yolk tube, and pericardial area of zebrafish is noticed when the developing zebrafish larvae were grown in a medium containing 70 mg L^−1^ as-prepared BaF_2_, BaF_2_:Ce^3+^/Tb^3+^, BaF_2_:Ce^3+^/Tb^3+^@SiO_2_, and BaF_2_:Eu^3+^nanoparticles. However, as the developing zebrafish larvae were kept in the medium of 0.1 mg L^−1^ cypermethrin pesticide, bending of the tail occurred due to deformation in skeletal muscles (Figure 12). (Sharma et al., 2020b).

**FIGURE 13:**
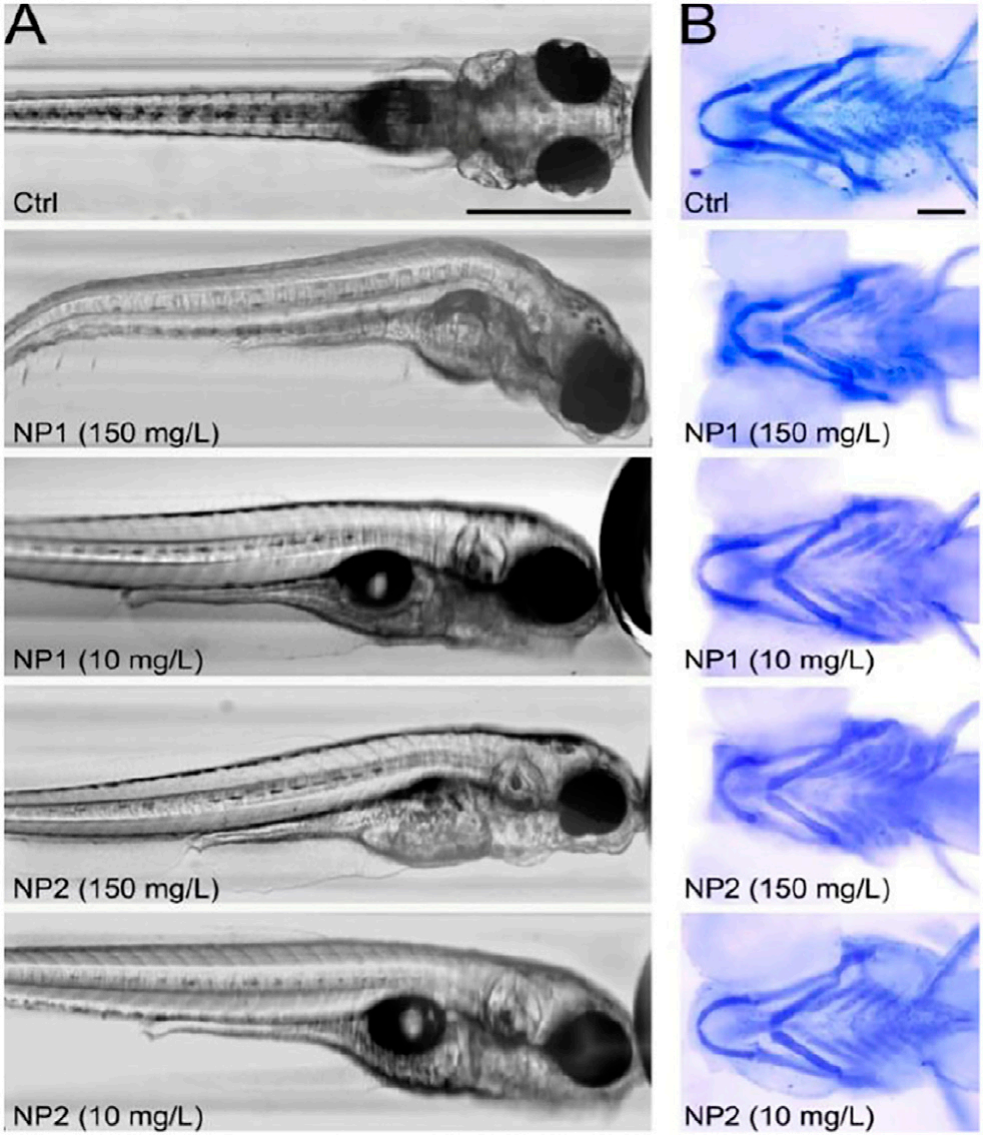
Morphological and anatomical abnormalities induced by nanoparticles. Representative bright-field images of different treatment groups showing **(A)** nearly complete larval body for morphological assessment; **(B)** craniofacial region for anatomical assessment. Scale bar: **(A)** 0.5 mm; **(B)** 0.2 mm (Chouryal et al., 2020).

In addition to the deformation in the skeletal muscles of the tail region (bright-field images, Figure 13 A), the effect of different concentrations of BaF_2_ (NP1) and B) BaF_2_:Ce^3+^/Tb^3+^ (NP2) nanoparticles on the craniofacial region of developing zebrafish larvae is also studied (Figure 13B). When developing zebrafish larvae were fixed and stained with alcian blue, normal craniofacial development of larvae is found in both the control and treated larvae with less (10 mgL^−1^) concentration of NP1 and NP2. Also, developed Meckel’s and ceratohyal cartilages are observed and analyzed (Figure 13B). Whereas by treating the larvae with high concentrations (150 mgL^−1^) of NP1 and NP2 nanoparticles, defects in the craniofacial region are observed, at low concentrations (10 mgL^−1^), such defects are not found. This study highlighted the required doses of nanoparticles for safer bioimaging applications (Chouryal et al., 2020).

### Bio-Molecule Detection

Functionalized Ln(s)-doped nanophosphors materials can also be utilized to sense bio-active molecules (Ju et al., 2013; Mi et al., 2013). In order to detect the presence of biomolecule in the solution, selectively biofunctionalizing the nanophosphors with suitable molecules like biotin is needed (Ju et al., 2013). Then, biotinylated nanophosphors can be used as a biosensor to detect the bio-molecules. The hydrophobic group containing the surface of nanophosphors is first functionalized with appropriate amphiphilic polymer in order to endow the surface with functional groups such as –COOH, –NH_2_, and –SH. As a result, the biocompatibility of nanophosphors is extensively improved for biosensing applications.

**FIGURE 14:**
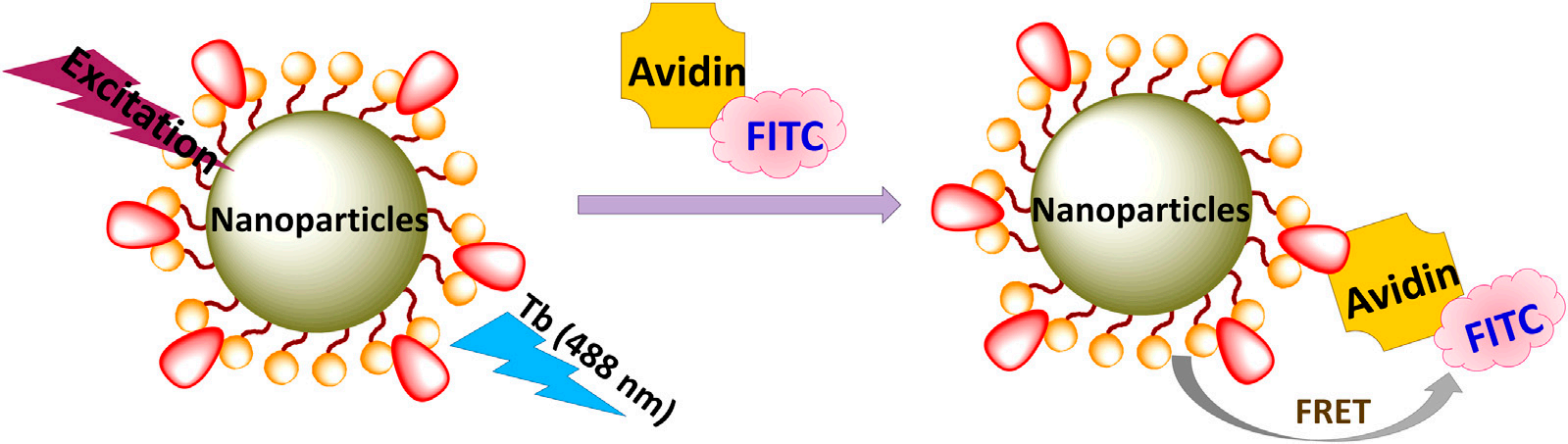
Schematic illustration of FRET detection of avidin.

For example, the surface of [P_66614_]^+^-capped NaGdF_4_:Ce, Tb nanocrystals was further modified with amphiphilic polymer ODA-PAA (octadecylamine modified polyacrylic acid) (Ju et al., 2013). Thereafter, the modified nanocrystals were biotinylated to detect the targeted molecule, i.e., FITC- (fluorescein isothiocyanate-) labeled avidin through a time-resolved fluorescence resonance energy (TR-FRET), as shown in Figure 14A. In the presence of biotinylated nanocrystals, emission of FITC was significantly increased due to the matching of emission band of Tb^3+^ at 488 nm with the absorption spectrum of FITC. Because of this interaction, when the concentration (nM) of FITC-labeled avidin into the system was increased, the emission intensity of Tb^3+^ (peak centered at 488 nm) was gradually found to be decreased (Figure 14B) (Ju et al., 2013) Additionally, LaF_3_:Ce,Tb nanoparticles were employed to detect the ascorbic acid in the range of 8.0 × 10^−6^ to 1.0 × 10^−4^ mol L^−1^. In the presence of ascorbic acid, the luminescence intensity of Tb^3+^ was quenched (Mi et al., 2013).

### Optical Sensor

**FIGURE 15:**
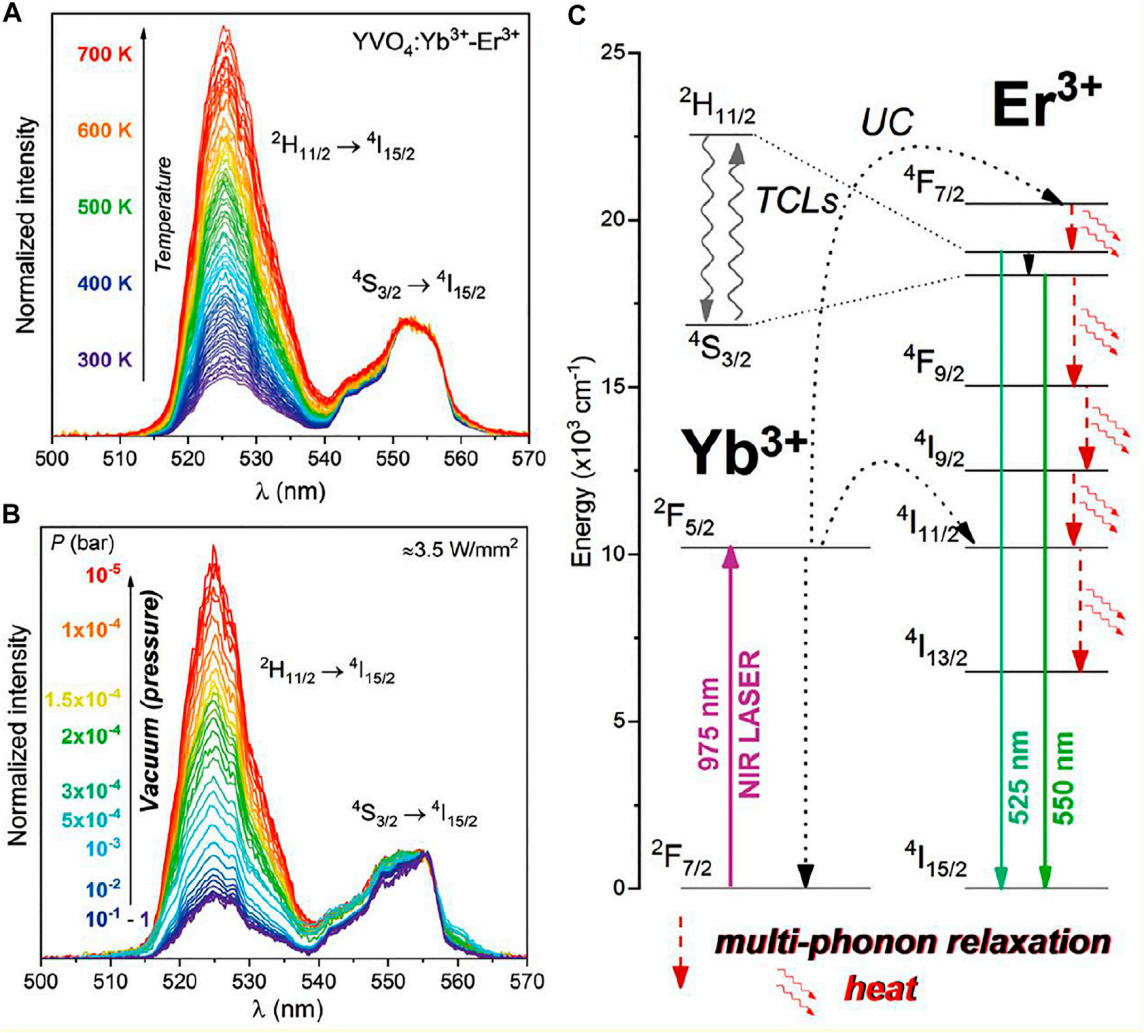
**(A)** UC emission spectra of the YVO_4_:Yb-Er sample, normalized to the ^4^S_3/2_ → ^4^I_15/2_ transition; λ_ex_ = 975 nm (≈0.5 W mm^−2^). **(B)** UC emission spectra recorded in the pressure range from ≈10^–5^ to 1 bar, with power density ≈3.5 W mm^−2^; λ_ex_ = 975 nm and **(C)** radiative and nonradiative processes occurring in the material studied, emphasizing thermalization of states and multiphonon relaxation (Runowski et al., 2020b).

In the last decade, RE-doped luminescent nanomaterials have been shown to be an excellent candidate for optical sensing applications like nanothermometry (sensing of alternation in temperature) and nanomanometry (sensing of pressure variation) (Runowski, 2020; Jaque and Vetrone, 2012). Interestingly, RE^+2/+3^ –doped nanomaterials show numerous pressure- and temperature-dependent changes in the spectral patterns, such as band ratio, spectral shift, intensity, bandwidth, and lifetimes. In the case of nanomanometry, when high pressure is applied to the materials, crystal lattice parameters, like bond length, unit cell volume, and symmetry of the local environment, are decreased due to compression. This leads to a change in luminescent patterns of the materials. Moreover, as the emission intensity of materials is highly sensitive to change in lattice parameters of the crystal, it is usually decreased with the pressure (Runowski, 2020; Jaque and Vetrone, 2012). In the case of nanothermometry, it is the remote, contactless, and high-resolution technique in which minute alternation in temperature brings about significant changes in the luminescence behavior of RE^3+^-doped nanomaterials. This technique is utilized for the optical sensing of temperature variation in extreme range (about ∼100 and ∼900 K) and can also be used to sense the changes in the biological range of temperature (∼290–330 K). For this application, several RE^+2/+3^ ions have been employed as dopant ions such as Pr^3+^, Nd^3+^, Yb/Tm^3+^, Yb^3+^/Er^3+^, Yb^3+^/Ho^3+^, Eu^2+^, Eu^3+^, Sm^3+^, Tb^3+^, and Er^3+^. The credit of thermal sensing by RE^3+^-doped (RE^3+^ = Nd^3+^, Er^3+^ or Tm^3+^) nanomaterials is given to the availability of the thermally coupled levels for which energy levels are separated in the range of 200–2000 cm^−1^ (Runowski, 2020; Jaque and Vetrone, 2012). For example, Ximendes et al. have applied the upconversion Er-Yb@Yb-Tm LaF_3_ core-shell nanoparticles for determining the properties of intrinsic subcutaneous tissues. This core-shell structure is used as an infrared luminescent nanothermometer to get insight into the heating and cooling effect on the luminescence of nanoparticles in the biological window and in this case, thermal sensitivities are obtained about 5% K^−1^ (Ximendes et al., 2017). Runowski et al. have prepared the optical vacuum sensor of upconverting materials YVO_4_:Yb^3+^,Er^3+^. In this study, they have depicted that the temperature-dependent emission band intensity ratio (525/550) of Er^3+^ TCLs can be used to get insight into local temperature. It has also been noticed that the laser-induced heating of the sample is significantly enhanced by 20 times in the vacuum. In other words, this luminescent material can be used for sensing the ultralow pressure even in the vacuum range (Figure 15). (Runowski et al., 2020b)

The upconverting NaGdF_4_:Yb^3+^(18%),Er^3+^(2%) microcrystals have been employed to study the luminescence thermometry in the range from ultralow (4 K) to room temperature (290 K). In this study, thermally coupled levels of Er^3+^ ions are used for ratiometric sensing from room temperature to 140 K (Mukhuti et al., 2020). Runowski et al. have prepared the Yb^3+^ and Tm^3+^ co-doped LaPO_4_ and YPO_4_ nanomaterials as a multifunctional optical sensor. These nanomaterials are employed as nanothermometers and nanomanometers to investigate the influence of temperature and pressure alterations on the emission of Tm^3+^ because of having large energy difference (1800 cm^−1^) between the thermalized states of Tm^3+^ which are highly sensitive to alterations of temperature (Runowski et al., 2018). As a result, Tm^3+^ band ratio of thermally induced transitions (^3^F_2,3_→^3^H_6_/^3^H_4_→^3^H_6_) is varied with temperature, which can explicitly be found in the emission spectra. They studied the effect of high pressure in the wide range (0.42–25.03 GPa for LaPO_4_:Yb,Tm; 0.64–24.35 GPa, YPO_4_:Yb,Tm) and temperature changes in the range of (293–773 K) on the luminescence and decay time of nanomaterials (Runowski et al., 2018).

## Conclusion and Future Outlook

In conclusion, room temperature task-specific ionic liquids (RTILs) are a versatile and tunable class of solvents that can be efficiently used in nanomaterials synthesis due to their interesting properties such low vapor pressure, large liquidus range, and tunability of its cation/anion combination. The discussion shows that ILs can be used not only as solvents and templating agents but also as reaction partners. This “all three-in-one” approach of ILs makes them superior to other conventional organic solvents. Various IL-assisted methodologies like solvothermal, microemulsion, sonication, and microwave methods can be used to prepare the crystal phase and size of nanoparticles and control their morphology. Combining the unique properties of ILs like high polarizability and conductivity with the fascinating aspects of microwave synthesis technique, a paradigm shift in nanomaterials synthesis can be achieved. Not only is reaction time reduced to a great extent (even in the level of seconds), but also the temperature impact of low temperature or metastable phases can be nicely explored.

On the other hand, RE^3+^-doped nanomaterials can be useful in several photonic and biophotonic applications due to their large Stokes shift, narrow emission band, and long decay time. Though the quantum confinement effect is not observed for dopant RE^3+^ ion, the luminescence property can be effectively tuned by varying the crystal phase, shape, size, and lattice strain of the host materials. Eventually, the above-mentioned properties of the host materials can be nicely tuned by changing the basic properties of ILs like cation/anion combinations, alkyl chain length, viscosity, and concentration of ILs. Thus, a good correlation can be made between luminescence dynamics of the dopant rare-earth ion inside the host materials and structural-physical properties of ionic liquids, which form the core of this review article. In addition, several photonic and biophotonic applications like white light-emitting materials, optical sensors for nanothermometry and nanomanometry, energy-efficient phosphor, FRET-based and other biological detections, *in vivo* and *in vitro* imaging are elaborated in this review article. In a nutshell, a sincere effort has been made to couple the basic principles of “green chemistry” of ILs with the interesting aspects of rare-earth-doped luminescent materials.

Despite substantial progress in ionic liquids, it is still a long way to go for successful applications in nanomaterials synthesis. One of the major drawbacks is preparing pure ILs, especially free of hydroxyl or water molecules. Secondly, it is very important to protect hydrophilic ILs from water molecules; therefore, researchers sometimes need to design reactions in an inert atmosphere or using glove boxes. Pertaining to biological applications of RE^3+^ doped upconverted materials, ultra-small nanoparticles are desired. However, common experience shows that UC efficiency significantly decreases with the decrease of particle size and also due to the deleterious surface quenching in colloidal dispersions. In addition, the UC quantum yield depends on excitation density and according to the American National Standard for Safe Use of Lasers, only a low irradiance of 0.1 W cm^−2^ (for 980 nm cw laser diode) can be applied to the human skin. Thus, it is a great challenge to the scientific community to realize an adequate upconversion efficiency under low power irradiation for *in vivo* studies. Though both the upconversion and quantum cutting downconversion materials have tremendous potential to increase the solar cell efficiency beyond the Schokley-Quiser efficiency limit, achieving high efficiency after making a device is still a big challenge. Last but not least, we believe that RE^3+^-doped luminescent nanomaterials *via* ionic liquids will continue to be a hotspot in research.
